# Composition, variation, expression and evolution of low-molecular-weight glutenin subunit genes in *Triticum urartu*

**DOI:** 10.1186/s12870-014-0322-3

**Published:** 2015-02-28

**Authors:** Guangbin Luo, Xiaofei Zhang, Yanlin Zhang, Wenlong Yang, Yiwen Li, Jiazhu Sun, Kehui Zhan, Aimin Zhang, Dongcheng Liu

**Affiliations:** State Key Laboratory of Plant Cell and Chromosome Engineering, National Center for Plant Gene Research, Institute of Genetics and Developmental Biology, Chinese Academy of Sciences, 1 West Beichen Road, Chaoyang District Beijing, 100101 China; University of Chinese Academy of Sciences, Beijing, 100049 China; College of Agronomy, The Collaborative Innovation Center of Grain Crops in Henan, Henan Agricultural University, 63 Nongye Road, Zhengzhou, 450002 China; Present address: Department of Agronomy & Plant Genetics, University of Minnesota, 1991 Buford Circle, St. Paul, MN 55108 USA

**Keywords:** Low-molecular-weight glutenin subunits, *Triticum urartu*, *Glu-A3*, Proteomics, Evolution

## Abstract

**Background:**

Wheat (AABBDD, 2n = 6x = 42) is a major dietary component for many populations across the world. Bread-making quality of wheat is mainly determined by glutenin subunits, but it remains challenging to elucidate the composition and variation of low-molecular-weight glutenin subunits (LMW-GS) genes, the major components for glutenin subunits in hexaploid wheat. This problem, however, can be greatly simplified by characterizing the LMW-GS genes in *Triticum urartu*, the A-genome donor of hexaploid wheat. In the present study, we exploited the high-throughput molecular marker system, gene cloning, proteomic methods and molecular evolutionary genetic analysis to reveal the composition, variation, expression and evolution of LMW-GS genes in a *T. urartu* population from the Fertile Crescent region.

**Results:**

Eight LMW-GS genes, including four m-type, one s-type and three i-type, were characterized in the *T. urartu* population. Six or seven genes, the highest number at the *Glu-A3* locus, were detected in each accession. Three i-type genes, each containing more than six allelic variants, were tightly linked because of their co-segregation in every accession. Only 2-3 allelic variants were detected for each m- and s-type gene. The m-type gene, *TuA3-385*, for which homologs were previously characterized only at *Glu-D3* locus in common wheat and *Aegilops tauschii*, was detected at *Glu-A3* locus in *T. urartu. TuA3-460* was the first s-type gene identified at *Glu-A3* locus. Proteomic analysis showed 1-4 genes, mainly i-type, expressed in individual accessions. About 62% accessions had three active i-type genes, rather than one or two in common wheat. Southeastern Turkey might be the center of origin and diversity for *T. urartu* due to its abundance of LMW-GS genes/genotypes. Phylogenetic reconstruction demonstrated that the characterized *T. urartu* might be the direct donor of the *Glu-A3* locus in common wheat varieties.

**Conclusions:**

Compared with the *Glu-A3* locus in common wheat, a large number of highly diverse LMW-GS genes and active genes were characterized in *T. urartu*, demonstrating that this progenitor might provide valuable genetic resources for LMW-GS genes to improve the quality of common wheat. The phylogenetic analysis provided molecular evidence and confirmed that *T. urartu* was the A-genome donor of hexaploid wheat.

**Electronic supplementary material:**

The online version of this article (doi:10.1186/s12870-014-0322-3) contains supplementary material, which is available to authorized users.

## Background

Wheat flour can be made into a wide variety of foods due to the unique viscoelastic properties of dough [1,2]. These viscoelastic properties result from gluten proteins, which account for about 80% of the total grain proteins [3,4]. Wheat gluten is composed of two main components: glutenin and gliadin. Glutenin plays a major role in dough’s elasticity, while gliadin contributes mainly to dough’s viscosity [5]. According to their relative mobility in sodium dodecyl sulphate polyacrylamide gel electrophoresis (SDS-PAGE), glutenin proteins are generally divided into high-molecular-weight glutenin subunits (HMW-GSs) and low-molecular-weight glutenin subunits (LMW-GSs) [6]. LMW-GSs have molecular weights ranging from 20 kDa to 45 kDa, making up 60% of glutenin proteins and one third of seed storage proteins [3,7]. Based on the first amino acid of the mature proteins, LMW-GSs have been classified into three types: i- (isoleucine), m- (methionine) and s- (serine) [8].

LMW-GSs are encoded by a multi-gene family whose members are located at *Glu-A3*, *Glu-B3* and *Glu-D3* loci on the short arms of homologous chromosomes 1A, 1B, and 1D, respectively [9]. Without a complete genome sequence, it is hard to determine the exact members of LMW-GS gene family in a wheat variety. In the past decade, the LMW-GS gene family members were characterized in only a few wheat varieties, including Norin 61, Glenlea and Xiaoyan 54 [10-12]. Twelve to 19 LMW-GS genes were identified from individual varieties using complementary methods, including cDNA or DNA BAC library screening and proteomic analysis. Recently, a new molecular marker system was developed to identify LMW-GS gene family members which used high-resolution capillary electrophoresis to separate fragments of gene members with three conserved primer sets (LMWGS1, LMWGS2 and LMWGS3) [13]. Using this marker system, more than 15 LMW-GS genes were detected from single wheat variety [13]. This marker system was also used as a complementary tool for the allelic determination of LMW-GS genes at *Glu-B3* locus in wheat cultivars and segregating populations [14]. A full-length gene-cloning method based on this marker system has been used to clone 16 or 17 LMW-GS genes in individual bread wheat genotypes [15]. Both the marker system and the gene cloning method were applied to investigate the composition of LMW-GS genes in large populations, including Aroona near-isogenic lines and the micro-core collections (MCC) of Chinese wheat germplasm [16,17], demonstrating their efficiency in dissecting this complex gene family in common wheat.

Wild progenitors and relatives could provide tremendous genetic variability to broaden the gene-pool of common wheat [18]. In the past decades, several important agronomic genes have been well characterized, such as the stem rust resistance gene *Sr47*, the leaf rust resistance genes (*Lr41*, *Lr42* and *Lr43*) from *Aegilops tauschii*, the high grain protein content (*Gpc-B1*) gene from tetraploid wheat and the chromosome arm 1RS containing both disease resistance and high yield genes from rye [19-22]. *T. urartu* is the wild diploid wheat from the Fertile Crescent region, and has long been considered as the A-genome donor in polyploid wheat species [23]. Isozyme, RAPD and AFLP markers have detected large genetic variations in *T. urartu* populations [24,25]. Recently, a set of genes were also characterized in *T. urartu*, e.g., the powdery mildew resistance gene (*PmU*), and the grain length controlling gene (*TuGASR7*) [26-28]. Abundant variability of storage proteins in *T. urartu*, was detected in gliadin proteins and HMW-GSs using electrophoretic procedures or nucleotide sequence analysis [29,30]. Several variants with repetitive domain length polymorphism were also observed in LMW-GS genes [31]. However, the detailed composition and genetic diversity of LMW-GS genes in *T. urartu* remain unknown.

Dissecting the composition and diversity of LMW-GS genes in *T. urartu* is prerequisite to broadening the genetic resources for bread-making quality improvement in common wheat; unraveling the genetic diversity of *T. urartu* will facilitate its gene and germplasm conservation. In this study, a systematic molecular analysis of LMW-GS genes in *T. urartu* was conducted using complementary approaches, including high-throughput molecular marker system, gene cloning, two-dimensional electrophoresis (2-DE), liquid chromatography tandem mass spectrometry (LC-MS/MS), matrix assisted laser desorption/ionization time of flight tandem mass spectrometry (MALDI-TOF/TOF-MS) and SDS-PAGE. The gene composition, variation, organization and expression pattern were extensively investigated in 157 accessions collected from the Fertile Crescent region, which is widely considered as the center of origin and diversity of *T. urartu* [25,32]. Genetic diversity of LMW-GS genes and genotypes in *T. urartu* and their evolutionary clues pertaining to wheat species of different ploidy were further discussed.

## Results

### Composition and variation of LMW-GS genes in *T. urartu*

**Figure 1:**
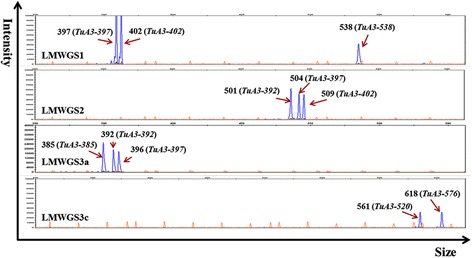
**Electropherograms of DNA fragments detected in accession PI428198 using the LMW-GS gene molecular marker system.** The horizontal axis shows the detected fragment sizes, and the vertical axis displays the signal intensities during the capillary electrophoresis. The orange peaks were size standard DNA fragments in the GeneScan 1200 LIZ and each blue peak represents a LMW-GS gene.

For each conserved primer set of the LMW-GS marker system [13], more than 16 DNA fragments were amplified from the *T. urartu* population. Totally, 25 non-redundant DNA fragments from the population, with six or seven from each accession, were determined and named according to the experimental or theoretical size of their corresponding fragments amplified by the LMWGS1 primer set (Table 1) [13,15,17]. Typically, the sequenced accession, PI428198 (G1812) [28], had seven LMW-GS genes, including *TuA3-385*, *TuA3-392*, *TuA3-397*, *TuA3-402*, *TuA3-520*, *TuA3-538* and *TuA3-576* (Figure 1; Table 1). Among 157 accessions, 15 different genotypes (U1-U15) were identified; each genotype had unique fragment sizes except for U5 and U6, which were discriminated by SNPs within three LMW-GS genes (*TuA3-502*, *TuA3-538* and *TuA3-576*) according to the subsequent gene cloning data (Table 2). Regarding the frequencies of the genotypes in the *T. urartu* population, U6 was the most abundant (39 accessions), followed by U2 (35 accessions), U10 (21 accessions) and U8 (16 accessions); the remaining 11 genotypes totally accounted for 24% of accessions, in which the genotypes U1, U7, U11 and U12 were discovered in only one or two accessions (Table 2).

**Table 1:** **LMW-GS genes and their allelic variants identified in** ***T. urartu*** **population using the LMW-GS gene molecular marker system**

**Gene**	**Allelic variants** ^**a**^				**LMWGS1** ^**b**^				**LMWGS2** ^**b**^				**LMWGS3** ^**b**^			
TuA3-385	TuA3-385				385				492				383			
TuA3-391	TuA3-373	TuA3-391	TuA3-392		373	391	392		480	484	501		371	375	390	
TuA3-397	TuA3-397				397				504				396			
TuA3-400	TuA3-400	TuA3-402			400	402			506	509			399	402		
TuA3-400	TuA3-460	TuA3-463	TuA3-474		460	N^c^	474		566	569	580		464	467	479	
TuA3-502	TuA3-495	TuA3-498	TuA3-502	TuA3-520	N	N	N	N	603	N	N	N	532	535	538	561
		TuA3-590	TuA3-593			N	N			N	N			633	636	
TuA3-538	TuA3-535	TuA3-538	TuA3-657		535	538	657		641	644	753		574	577	697	
TuA3-576	TuA3-406	TuA3-555	TuA3-576	TuA3-579	N	555	576	N	517	N	682	685	443	597	618	621
		TuA3-597	TuA3-669			597	669			N	773			640	720	

^a^A single gene could be detected by no less than one primer set, and the correspondence among fragments detected by these three primer sets was established by their theoretical sizes. LMW-GS genes and allelic variants were named in accordance with the sizes of their corresponding fragments amplified by the LMWGS1 primer set practically or theoretically, and the major allelic variant was designated as the gene whereas the remainders as its allelic variants [17].

^b^Three primer sets of the LMW-GS gene molecular marker system [13].

^c^Not amplified by the specific primer sets.

**Table 2:** **Genotypes and geographic distribution of LMW-GS genes in** ***T. urartu*** **population**

**Genotype**	**m-type**				**s-type**	**i-type**			**Original region** ^**#**^						**Accession number**
	**TuA3-385**	**TuA3-391**	**TuA3-397**	**TuA3-400**	**TuA3-460**	**TuA3-502**	**TuA3-576**	**TuA3-538**	**Iraq**	**Iran**	**Armenia**	**Lebanon**	**Syria**	**Turkey**	
**U1**	TuA3-385a^+^	TuA3-373	TuA3-397a			TuA3-502c^*^	TuA3-576d	TuA3-538a^*^						1	1
**U2**	TuA3-385b	TuA3-391	TuA3-397a			TuA3-502b	TuA3-406	TuA3-538c^*^	1	1		9	1	23	35
**U3**	TuA3-385a	TuA3-391	TuA3-397a			TuA3-498^*^	TuA3-597^*^	TuA3-535^*^						5	5
**U4**	TuA3-385b	TuA3-392	TuA3-397a			TuA3-520^*^	TuA3-576c^*^	TuA3-538d						7	7
**U5**	TuA3-385a	TuA3-392	TuA3-397a			TuA3-502c^*^	TuA3-576b^*^	TuA3-538b^*^						9	9
**U6**	TuA3-385a	TuA3-392	TuA3-397a			TuA3-502a^*^	TuA3-576a^*^	TuA3-538a^*^				36	2	1	39
**U7**	TuA3-385a	TuA3-392	TuA3-397a			TuA3-502d	TuA3-555^*^	TuA3-538a^*^				1		1	2
**U8**	TuA3-385a	TuA3-392	TuA3-397b^*^			TuA3-502a^*^	TuA3-579b^*^	TuA3-538b^*^				15	1		16
**U9**	TuA3-385a	TuA3-392	TuA3-397a			TuA3-593	TuA3-576b^*^	TuA3-538e^*^				1	1	4	6
**U10**	TuA3-385a	TuA3-373	TuA3-397a		TuA3-460	TuA3-502a^*^	TuA3-579a^*^	TuA3-538b^*^			1	19		1	21
**U11**	TuA3-385a	TuA3-373	TuA3-397a		TuA3-463	TuA3-520^*^	TuA3-576c^*^	TuA3-538a^*^						2	2
**U12**	TuA3-385b	TuA3-373	TuA3-397a		TuA3-463	TuA3-495	TuA3-669^*^	TuA3-657^*^						1	1
**U13**	TuA3-385a	TuA3-392	TuA3-397a		TuA3-474	TuA3-502c^*^	TuA3-576d	TuA3-538d				1		3	4
**U14**	TuA3-385a	TuA3-392	TuA3-397a	TuA3-402		TuA3-520^*^	TuA3-576c^*^	TuA3-538a^*^						5	5
**U15**		TuA3-392	TuA3-397b^*^	TuA3-400^*^		TuA3-590	TuA3-576e^*^	TuA3-538a^*^			4				4

^+^Letter following the number is used to distinguish allelic variants with the same size of DNA fragment.

^*^Asterisks label active genes with their protein products detected in SDS-PAGE or 2-DE.

^#^Lebanon and Turkey represent Northeastern Lebanon and Southeastern Turkey, respectively.

To further characterize the LMW-GS genes represented by these DNA fragments, 50 typical accessions, covering all 15 genotypes, were subjected to gene cloning using the full-length gene cloning method (Additional file 1: Table S1) [15]. Generally, six or seven LMW-GS gene sequences were cloned in each accession, which matched well with six or seven DNA fragments detected with the marker system. Totally, 148 LMW-GS sequences were obtained and deposited in GenBank (KM065455-KM065457, KM085178-KM085322); these sequences were derived from eight LMW-GS genes (i.e., *TuA3-385*, *TuA3-391*, *TuA3-397*, *TuA3-400*, *TuA3-460*, *TuA3-502*, *TuA3-538* and *TuA3-576*) determined in the *T. urartu* population due to the redundancy and large number of allelic variants (Table 2). Among these genes, only two or three variants were detected for each of the *TuA3-385*, *TuA3-391*, *TuA3-397, Tu-A3-400* and *TuA3-460* genes. In contrast, at least seven variants were identified for each of the *TuA3-502*, *TuA3-538* and *TuA3-576* (Table 2). All allelic variants resulted in 15 genotypes at the *Glu-A3* locus in *T. urartu*, which was consistent with the genotypes based on the size of DNA fragment in the marker system (Table 2).

### LMW-GS genes in *T. urartu*

Among the eight genes, four (*TuA3-385*, *TuA3-391*, *TuA3-397* and *TuA3-400*) were m-type, three (*TuA3-502*, *TuA3-538* and *TuA3-576*) were i-type and one (*TuA3-460*) was s-type.

### m-type LMW-GS genes

*TuA3-385* gene with two variants, *TuA3-385a* and *TuA3-385b*, was widely distributed in the *T. urartu* population. Both variants were supposed as pseudo-genes due to immature stop codons at their repetitive domains (Additional file 1: Table S2; Additional file 2: Figure S1). Another common gene, *TuA3-391* contained three allelic variants: *TuA3-373*, *TuA3-391* and *TuA3-392* (Additional file 1: Table S2; Additional file 2: Figure S2). All three allelic variants of *TuA3-391* gene were pseudo-genes because of immature stop codons at either their repetitive or C-terminal I domains. *TuA3-397* was also a common gene in *T. urartu* population. *TuA3-397a* was the major allelic variant (87.26%), but it might be a pseudo-gene. The other variant, *TuA3-397b* might encode an m-type LMW-GS for its intact open reading frame (ORF) (Additional file 1: Table S2; Additional file 2: Figure S3). *TuA3-400* gene was seldom detected in *T. urartu* population; its two allelic variants (*TuA3-400* and *TuA3-402*) were only detected in four and five accessions, respectively (Additional file 1: Table S2). These two allelic variants shared 99% identity despite two 3-bp InDels and several SNPs (Additional file 2: Figure S3). The *TuA3-402* allelic variant was a pseudo-gene due to the immature stop codon at its C-terminal II domain, whereas *TuA3-400* was supposed to be active for its intact ORF.

### i-type LMW-GS genes

Three i-type genes: *TuA3-502*, *TuA3-538* and *TuA3-576* were identified in each *T. urartu* accession. The *TuA3-502* gene had nine variants: *TuA3-495*, *TuA3-498*, *TuA3-502a/b/c/d*, *TuA3-520*, *TuA3-590* and *TuA3-593* (Additional file 2: Figure S4). *TuA3-502a* was the major allelic variant of the *TuA3-502* gene with an occupation of 48.41% accessions, and *TuA3-502b* was another widely distributed variant and detected in 22.29% accessions (Additional file 1: Table S2). Among nine variants, only *TuA3-498*, *TuA3-502a* and *TuA3-502c* might be active genes with intact ORFs.

Another i-type gene *TuA3-538* had seven variants: *TuA3-535*, *TuA3-538a/b/c/d/e* and *TuA3-657* in the *T. urartu* population. All the variants were supposed to be active genes for their intact ORFs, except for the *TuA3-538d* variant. The *TuA3-538a/b/c/d/e* variants distinguished themselves mainly by different repeat number of CAG and CAA motifs at the C-terminal II domain in addition to several SNPs throughout their coding regions. The *TuA3-535* variant shared >99% identity with each of the *TuA3-538a/b/c/d/e* variants. Compared with the other allelic variants of the *TuA3-538* gene, the long fragment of *TuA3-657* was mainly derived from two insertions (24-bp and 87-bp) at the repetitive domain (Additional file 2: Figure S5). Among these allelic variants, the *TuA3-538a, TuA3-538b* and *TuA3-538c* were widely distributed in the *T. urartu* population, occupying 85.35% of variants. *TuA3-535*, *TuA3-538d*, *TuA3-538e* and *TuA3-657* were rare, present in a few accessions (Additional file 1: Table S2).

The *TuA3-576* gene contained 11 variants: *TuA3-406*, *TuA3-555*, *TuA3-576a/b/c/d/e*, *TuA3-579a/b*, *TuA3-597* and *TuA3-669*. Many of them might be active genes based on their intact ORFs, whereas *TuA3-406* and *TuA3-576d* were pseudo-genes with immature stop codons at their repetitive and C-terminal II domains. The *TuA3-576a/b/c/d/e* variants were distinguished by InDels at C-terminal II domain and SNPs throughout their coding sequences (Additional file 2: Figure S6). Long deletions and insertions caused different fragment lengths of the variants of *TuA3-576* gene. Two deletions, 142-bp at the repetitive domain and 30-bp at the C-terminal I domain, were detected in the *TuA3-406* variant. A 24-bp deletion was also found at the repetitive domain of *TuA3-555* variant. In the *TuA3-597* variant, a 24-bp insertion was identified at its repetitive domain. Three insertions were detected in the *TuA3-669* variant: 33-bp and 69-bp at the repetitive domain, and 21-bp at the C-terminal III domain (Additional file 2: Figure S6). Among these variants, the *TuA3-576a, TuA3-406*, *TuA3-579a*, *TuA3-579b* and *TuA3-576b* were widely distributed in the *T. urartu* population, with proportions of 24.84%, 22.29%, 13.38%, 10.19% and 9.55%, respectively (Additional file 1: Table S2).

### s-type LMW-GS gene

TuA3-460 has the N-terminal region (MENSHIPGLEKPS) of typical s-type LMW-GS and a short s-type protein specific peptide (TLSH) at the repetitive domain (Additional file 2: Figure S7). The first amino acid of the mature protein of TuA3-460 was Ser after the peptide MEN were cut from the original protein. Thus, TuA3-460 belonged to s-type LMW-GS. The *TuA3-460* gene was the only s-type LMW-GS gene detected in the *T. urartu* population. Its three variants: *TuA3-460*, *TuA3-463* and *TuA3-474*, shared >99% identity. And all were pseudo-genes with immature stop codons both at their repetitive and C-terminal I domains. Compared with *TuA3-460,* the 3-bp (CCA) and 12-bp (CAACAACAACAA) insertions at their repetitive domains were responsible for the larger fragment lengths of *TuA3-463* and *TuA3-474,* respectively (Additional file 2: Figure S8). The *TuA3-460* gene was detected in only 17.80% accessions, *TuA3-460* (21 accessions), *TuA3-463* (3 accessions), and *TuA3-474* (4 accessions) (Additional file 1: Table S2)*.*

### Expression of LMW-GS genes in *T. urartu*

**Figure 2:**
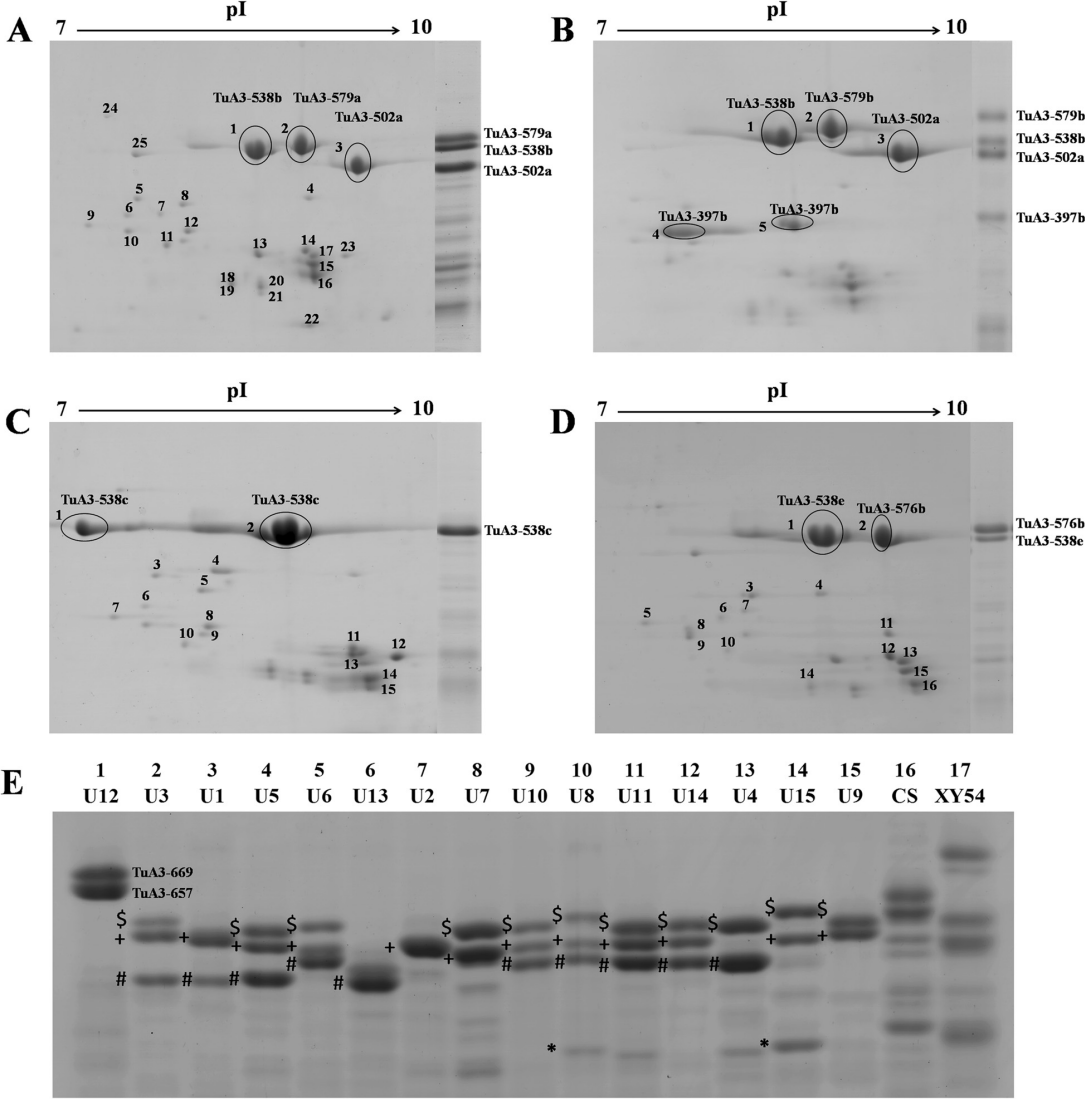
**Separation and identification of LMW-GS proteins in** ***T. urartu*** **using 2-DE and SDS-PAGE. (A-D)** are 2-DE (left) and SDS-PAGE (right) gels of PI428270 (U10), PI428335 (U8), PI428202 (U2), and PI428255 (U9); LMW-GS protein spots are circled in 2-DE gels. **(E)** is the SDS-PAGE banding pattern of LMW-GS proteins in 15 genotypes of *T. urartu* and two representatives of common wheat: Chinese spring (CS) and Xiaoyan54 (XY54). *, #, + and $ denote protein bands of active allelic variants of *TuA3-397*/*TuA3-400*, *TuA3-502*, *TuA3-538* and *TuA3-576* genes, respectively.

The bread-making quality of wheat flour is attributed greatly to the composition of LMW-GSs and the number of expressed genes [12,16]. To investigate the expression pattern of LMW-GS genes in *T. urartu,* four accessions from four genotypes, U2 (PI428202), U9 (PI428255), U10 (PI428270) and U8 (PI428335), in turn containing one, two, three and four genes with intact ORFs, were selected and subjected to proteomic analysis. All the spots on 2-DE gels of PI428202, PI428255 and PI428270, and the spots of LMW-GSs of PI428335 were identified by LC-MS/MS or MALDI-TOF/TOF MS (Figure 2).

Of the 25 spots investigated for PI428270 in the U10 genotype, three (spots 1, 2 and 3) were LMW-GSs, two were globulin, 13 were gliadins, and the remaining spots were other storage proteins (avenin, hordein and avenin-like precursor) (Figure 2a; Additional file 1: Table S3, S4 and S5). Spots 1, 2 and 3 were in turn matched to protein TuA3-538b, TuA3-579a and TuA3-502a in U10, and corresponded to the middle, upper and lower bands in SDS-PAGE due to their same mobility, respectively (Figure 2a; Additional file 1: Table S3). In the PI428335 accession with the U8 genotype, five spots were identified as LMW-GSs. Spots 1, 2 and 3 in turn matched deduced amino acid sequences of *TuA3-538b*, *TuA3-579b* and *TuA3-502a*, whereas both spots 4 and 5 matched *TuA3-397b*. All of these spots had corresponding bands with the same mobility in SDS-PAGE (Figure 2b; Additional file 1: Table S3). Interestingly, TuA3-400, which was only identified in the U15 genotype, shared the same 2-DE protein spot and SDS-PAGE band with TuA3-397b due to their similar molecular mass and isoelectric point (pI) value in our previous MS/MS identification (Data not shown) (Table 2; Figure 2e). In PI428202, spots 1 and 2 were proteins of the only active variant, *TuA3-538c* in U2; six (spot 1) and eight (spot 2) high-quality peptide sequences obtained by MS/MS analysis matched hypothetical polypeptides of *TuA3-538c*, respectively (Figure 2c; Additional file 1: Table S3). Moreover, these two spots also corresponded to the only band (TuA3-538c) detected with SDS-PAGE (Figure 2c). With regard to PI428255 of the U9 genotype, spot 1 was a protein product of the *TuA3-538e* variant in U9 and corresponded to the lower band (TuA3-538e) in SDS-PAGE, and spot 2 was that of the *TuA3-576b* variant and matched the upper band (TuA3-576b) in SDS-PAGE (Figure 2d; Additional file 1: Table S3). The SDS-PAGE data for all the genotypes confirmed that four main types of bands corresponded to intact ORFs of *TuA3-397*/*TuA3-400*, *TuA3-502*, *TuA3-538* and *TuA3-576* in this *T. urartu* population by comparing their electrophoretic mobility with deduced protein molecular weights (Figure 2e). Collectively, the expression patterns of LMW-GS genes in *T. urartu* were consistent with the active genes determined using the LMW-GS marker system and full-length gene cloning method.

Generally, i-type genes were the main active genes in *T. urartu*, and one to three of them were expressed in individual accessions (Table 2, Figure 2e). One i-type variant, *TuA3-538c*, was expressed in 35 accessions of the U2 genotype. And all three i-type genes, *TuA3-502*, *TuA3-538* and *TuA3-576* were characterized as expressed genes in the U3, U5, U6, U10, U8, U11 and U14 genotypes, which together contained 61.78% of the total accessions (Table 2). All the m-type genes were pseudo-genes except for the *TuA3-397b* and *TuA3-400* allelic variants, which were only detected in the U8 (*TuA3-397b*) and U15 (*TuA3-397b* and *TuA3-400*) genotypes (Table 2, Figure 2e). None of the variants of the s-type gene, *TuA3-460*, were active as no protein bands were detected on 2-DE and SDS-PAGE, which was consistent with the stop codons in their CDS regions (Table 2, Figure 2e).

### Characteristics of LMW-GS genes in *T. urartu*

Based on the first amino acid of their mature protein sequences, the eight genes in *T. urartu* were classified into three types (m-, s- and i-). *TuA3-385*, *TuA3-391*, *TuA3-397* and *TuA3-400* were m-type, *TuA3-460* was s-type and *TuA3-502*, *TuA3-538* and *TuA3-576* were i-type genes. Their deduced mature proteins contained three conserved domains (N-terminal domain, repetitive domain and C-terminal domain), except for the i-type subunit which lacked the N-terminal domain (Additional file 2: Figure S9). Cysteine residues could form inter- and intra-chain disulphide bonds which are of great importance for the formation of glutenin polymers [3]. All the subunits identified in the *T. urartu* population contained eight cysteine residues. The location of these cysteine residues, in the m- and i-type genes, were conserved with six of the residues at the C-terminal I domain and one at each of the C-terminal II and III domains, except for the first and the third cysteine residues in the m-type genes, TuA3-397b and TuA3-400 (Additional file 2: Figure S9). The m-type LMW-GSs were also different from the i-type genes in molecular weight. The estimated molecular weight of TuA3-397b and TuA3-400 were 31.77 kDa and 31.90 kDa, respectively, substantially lower than the average molecular weight of all the i-type genes (TuA3-502, 36.98 kDa; TuA3-538, 38.55 kDa; TuA3-576, 39.42 kDa) because of longer repetitive regions in the i-type subunits (Additional file 2: Figure S9).

Among the three i-type genes, *TuA3-502* was more tightly linked with *TuA3-576* gene than *TuA3-538* gene, since a set of variants of *TuA3-502* and *TuA3-576* genes co-segregated (e.g., *TuA3-520* co-occurred with *TuA3-576c* in U4, U11 and U14 genotypes, *TuA3-590* was coupled with *TuA3-576e* in U15 genotype and *TuA3-520a* co-occurred with *TuA3-576a*, *TuA3-579a* and *TuA3-579b* in U6, U10 and U8 genotypes, respectively.) (Table 2). Interestingly, the *TuA3-502b*, *TuA3-406* and *TuA3-538c* variants might form a haplotype (*TuA3-502b/TuA3-406/TuA3-538c*), because they co-segregated exclusively in 35 accessions of the U2 genotype (Table 2). *TuA3-498*, *TuA3-535* and *TuA3-597* might also form a haplotype due to their co-occurrence in the five accessions of the U3 genotype (Table 2).

**Figure 3:**
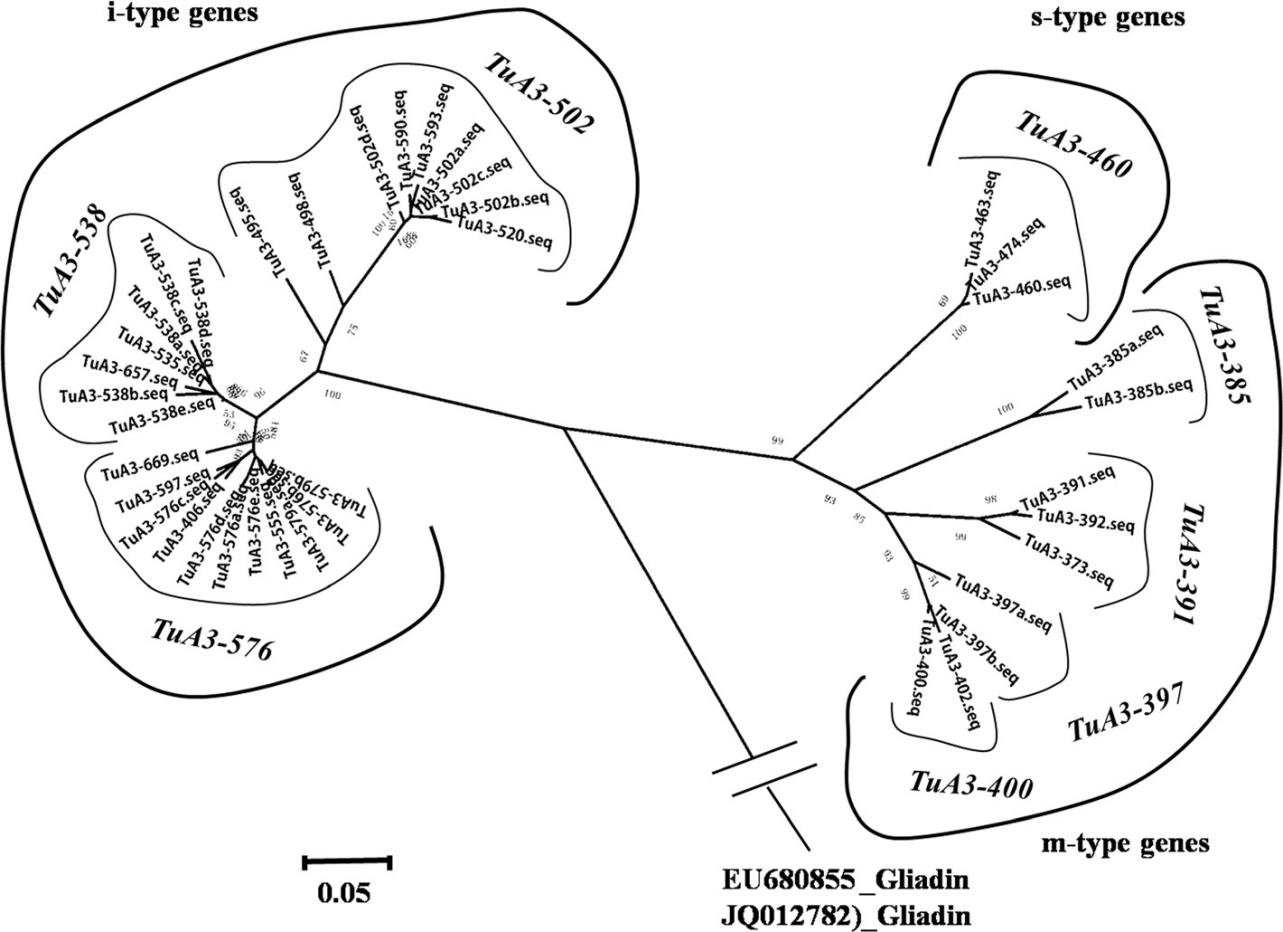
**Phylogenetic reconstruction of all the LMW-GS genes and their allelic variants identified in the** ***T. urartu*** **population.** All LMW-GS genes were divided into three groups, consistent with the i-, s- and m-type genes.

All variants of the eight LMW-GS genes in *T. urartu* were subjected to phylogenetic analysis using ClustalW2 and MEGA 5. Two main clades were obtained in the phylogenetic tree, one containing all the m- and s-type genes, and the other including all the i-type genes (Figure 3). In the m- and s-type gene clade, four sub-clades were further divided, each containing variants of a single gene, except for the sub-clade of *TuA3-397*, where *TuA3-400* was also involved (Figure 3). In the clade of i-type genes, three sub-clades were further divided, which corresponded to the *TuA3-502*, *TuA3-538* and *TuA3-576* genes, accordingly (Figure 3).

### Geographic distribution of LMW-GS genes and genotypes in *T. urartu*

The 157 analyzed *T. urartu* accessions were collected in the Fertile Crescent region, including northeastern Lebanon, southeastern Turkey, Armenia, Syria, Iraq and Iran, where many temperate-zone agricultural crops originated and were domesticated [33]. For the purpose of better exploitation and in situ genetic conservation of *T. urartu* germplasm, the geographic distribution of their LMW-GS genes/variants and genotypes was analyzed.

**Figure 4:**
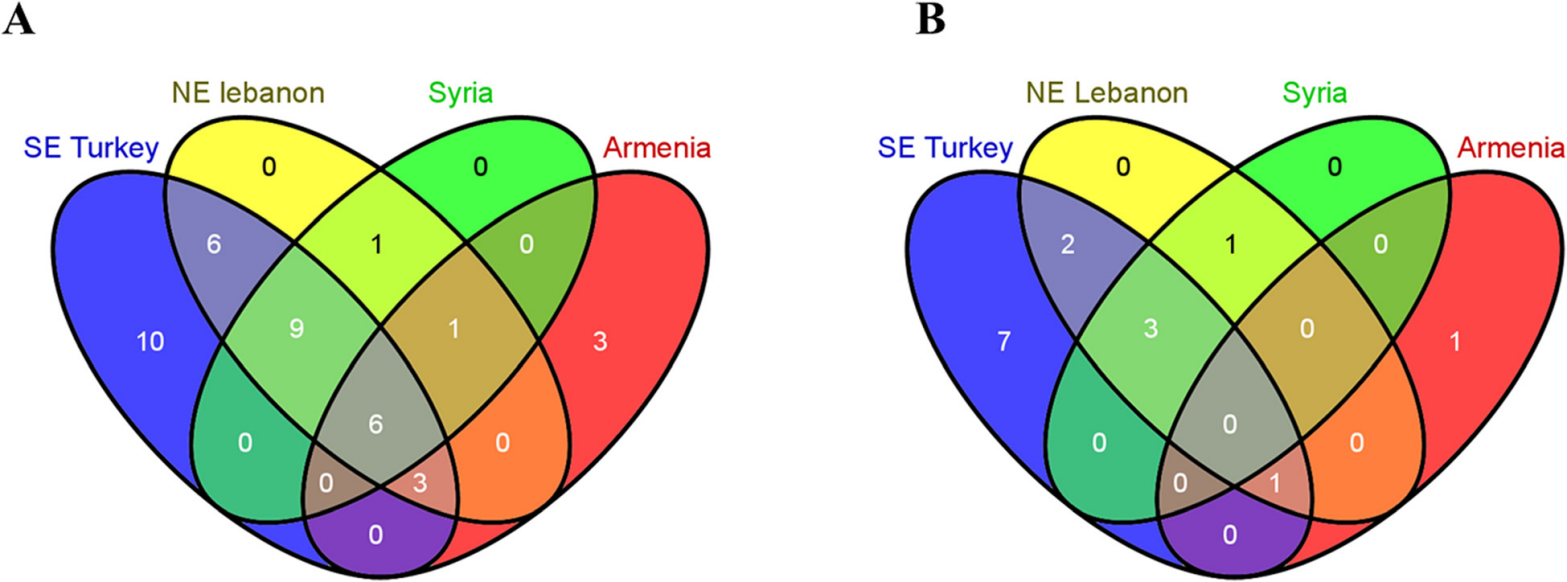
**Geographic distribution of LMW-GS genes/allelic variants and genotypes in** ***T. urartu*** **. (A)** Geographic distribution of LMW-GS genes/allelic variants. **(B)** Geographic distribution of LMW-GS genotypes. Iraq and Iran were not considered for only one accession was collected in each. SE Turkey stands for southeastern Turkey, and NE Lebanon for northeastern Lebanon.

Southeastern Turkey was the region of the greatest diversity where all eight genes and 34 of their total 39 variants were detected, as well as ten unique variants were found (Table 2, Figure 4a). In northeastern Lebanon, 26 variants of seven genes (all except *TuA3-400*) were detected; all were shared by southeastern Turkey except *TuA3-397b* and *TuA3-579b* (Table 2, Figure 4a). With regard to the genotypes of LMW-GS genes, southeastern Turkey was also the region of the highest/(most abundant) diversity, as the majority of genotypes were detected there (Table 2, Figure 4b). Moreover, seven genotypes (U1, U3, U4, U5, U11, U12 and U14) were unique to southeastern Turkey (Table 2, Figure 4b). In northeastern Lebanon and Syria, seven and four genotypes were detected, respectively. All genotypes were also present in southeastern Turkey except for the U8 genotype (Table 2, Figure 4b). Despite containing the unique genotype, U15, Armenia shared the U10 genotype with southeastern Turkey and northeastern Lebanon (Table 2, Figure 4b). In Iraq and Iran, the only genotype, U2, was also detected in southeastern Turkey, northeastern Lebanon and Syria (Table 2, Figure 4b). In summary, southeastern Turkey should be the center of origin for *T. urartu* because the greatest diversity of LMW-GS genes/variants and genotypes were detected. And in the five remaining collection areas, almost all the genes/variants and genotypes were observed in southeastern Turkey.

## Discussion

### LMW-GS genes in *T. urartu*

Eight LMW-GS genes, i.e., four m-type, three i-type and one s-type genes, were characterized in the *T. urartu* population. In each accession, six to seven genes were detected, the highest number of LMW-GS genes reported at *Glu-A3* locus to our best knowledge. To investigate the evolutionary relationships of LMW-GS genes between *T. urartu* and other diploid or polyploid wheats, all the gene sequences in *T. urartu* were queried with the nucleotide BLAST program in NCBI. Gene sequences sharing high identity (>90%, even 99%) with the LMW-GS genes in *T. urartu* were found (Additional file 1: Table S6).

**Figure 5:**
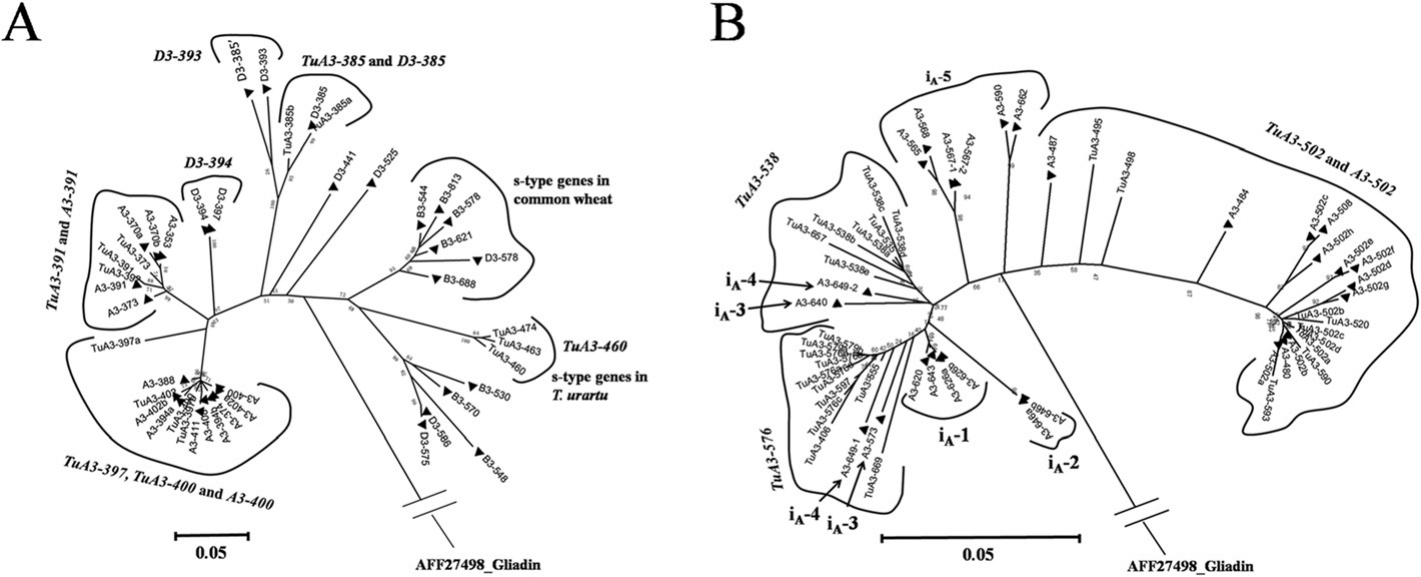
**Phylogenetic analysis of deduced protein sequences of LMW-GS genes in** ***T. urartu*** **and common wheat. (A)** Phylogenetic analysis of m- and s-type genes. **(B)** Phylogenetic analysis of i-type genes. Triangle represents sequences from common wheat. The gliadin protein sequence (AFF27498) was used as the out-group.

As the homolog of *TuA3-391* gene, *A3-391* was previously identified in common wheat [17]. This gene was extremely conserved between *T. urartu* and common wheat with 99% identity shared among its variants. (Additional file 1: Table S6). *TuA3-397* was universal in *T. urartu*. Its variants, *TuA3-397a* and *TuA3-397b,* shared more than 96% identity with *A3-394*b and *A3-400*, allelic variants of *A3-400* gene in common wheat, respectively (Additional file 1: Table S6) [17]. *TuA3-397* and *A3-400* should be homologs. Another gene, *TuA3-402* in *T. urartu,* also showed 99% identity with variants of *A3-400* gene (Additional file 1: Table S6). *TuA3-402* was only detected in U14 and U15 whereas *TuA3-397* was a common gene in *T. urartu*. Moreover, both genes were located in the same branch in the phylogenetic tree (Figure 5a). It might be reasonable to hypothesize that the *TuA3-402* gene was derived from a duplication of the *TuA3-397* gene.

For *TuA3-385*, no homolog was found at *Glu-A3* locus of other wheat species, but interestingly this gene shared >98% identity with *D3-385* at the *Glu-D3* locus (e.g., JX878094) in hexaploid wheat [17] and the *GluDt-64* allele (EF437430) in *Ae. tauschii* (Additional file 1: Table S6). And phylogenetic analysis showed the *TuA3-385* and *D3-385* genes clustered together (Figure 5a). Thus, the *TuA3-385* and *D3-385* genes were homologs between *T. urartu* and common wheat. However, *TuA3-385* was a pseudo-gene and *D3-385* was an active gene in both common wheat and *Ae. tauschii*. This m-type gene should be an ancient gene that emerged before the divergence of the A and D genomes, but this gene was lost at the *Glu-A3* locus during wheat polyploidization (Additional file 1: Table S6; Additional file 2: Figure S1). During evolution, this gene in *T. urartu* was mutated and became a pseudo-gene, but the gene in *Ae. tauschii* and common wheat maintained an intact ORF.

*TuA3-460* was the first s-type LMW-GS gene detected at the *Glu-A3* locus in *Triticum*. Interestingly, all of its BLAST hits (≤90% identities) in BLAST databases were not s-type but m-type genes (Additional file 1: Table S6). And the phylogenetic analysis indicated that the variants of *TuA3-460* were in the same clade with m-type genes at the *Glu-B3* and *Glu-D3* loci in *Triticum aestivum* (*B3-530*, *B3-578*, *B3-570*, *D3-575* and *D3-586*) and *Ae. speltoides* (FJ824794) but not s-type genes (Figure 5a). Protein sequence alignments were performed to understand the variations among TuA3-460, the known s- and m-type LMW-GSs (Additional file 2: Figure S7). Besides the conserved s-type N-terminal domain (MENSHIPGLEKPS) and the s-type specific peptide (TLSH) at the repetitive domain, TuA3-460 had 11 unique amino acids throughout the protein sequences, including one unique Cysteine residue at the C-terminal II domain. Moreover, compared with the known m- and s-type LMW-GSs, TuA3-460 contained three deletions (PPFSQQ, PVLPQQ and PPFSQQQQ) at the repetitive domain (Additional file 2: Figure S7). Thus, *TuA3-460* is a new s-type gene at the A genome, which is homologous with the s-type genes at the B and D genomes, rather than a chimeric gene containing s- and m-type gene sequences. The s-type LMW-GS genes were closer to the m-type genes than the i-type ones (Figure 3). Our deduced protein sequence alignments also revealed that the s-type LMW-GSs had higher similarities with the m-type LMW-GSs (Additional file 2: Figure S9), and the m-type LMW-GSs shared the variations with all s-type proteins from A, B and D genomes. Thus, m-type gene should be the oldest type of LMW-GS gene, and s-type genes probably originated from m-type LMW-GS genes due to the mutation of MET to MEN in the N-terminal region, which was consistent with the previous observations [3,17,34]. Even though containing several unique features, TuA3-460 had a pretty high similarity with the m-type genes, especially possessed one insertion (KQLGQCSFQQPQQQ) at the C-terminal domain and four amino acids (Additional file 2: Figure S9), which were exclusively contained in the m-type LMW-GSs. All these data indicate that this new s-type TuA3-460 gene also originated from the m-type LMW-GS genes. However, most features (specific amino acids and InDels) of the previously characterized s-type LMW-GSs could not be detected in TuA3-460, implying that *TuA3-460* might not share the same evolutionary process with other s-type LMW-GS genes from the primitive m-type LMW-GS gene, or they could originate from different m-type genes.

Three i-type genes, *TuA3-502*, *TuA3-538* and *TuA3-576* detected in *T. urartu* were relatively conserved across *Triticum* species. All variants of the *TuA3-502* gene share high identity (≥95%) with the *A3-502b* allele (JX877857) in common wheat, except for *TuA3-498* and *TuA3-593*, which were homologous to *A3-502f* (JX878133) (93% identity) and *A3-484* (JX878099) (94% identity), respectively (Additional file 1: Table S6. Many variants of the *TuA3-538* gene showed higher identity (>97%) with *A3-649-2* and *A3-640* than the other i-type genes in common wheat. The *TuA3-576* gene in *T. urartu* might be the homolog of *A3-649-1* and *A3-573* in common wheat due to their high identity (>97%). The variants of these three i-type genes also showed high identities to i-type genes identified in wheat relatives, with *TuA3-502c* to AJ293098 (98% identity) in *Triticum durum*, *TuA3-538b* to FJ441107 (95% identity) in *Triticum monococcum* and *TuA3-576a* to DQ217661 (93% identity) in *Triticum dicoccoides* (Additional file 1: Table S6).

However, the i-type genes preserved high polymorphisms at the *Glu-A3* locus of *Triticum* [17]. In *T. urartu*, all accessions possessed three i-type genes, compared with 2-4 genes in common wheat, implying that the *Glu-A3* locus might be derived from more than one origin of *T. urartu* and suffered rapid genome divergence. Moreover, all the three i-type genes in many *T. urartu* accessions were active genes, while only one or two genes were expressed in common wheat. This indicated that *T. urartu* is valuable in quality improvement in common wheat since a high number of active genes might contribute to superior bread-making quality [12]. Moreover, the i-type genes (*A3-502*/*A3-573*/*A3-640*, *Glu-A3f*) had positive effects on dough quality, e.g. percentage of SDS-unextractable fraction in total polymeric protein, dough resistance and extensibility [16]. The corresponding homologs of *A3-573* and *A3-640* were also detected in *T. urartu*.

### Center of origin and diversity of *T. urartu*

Turkey was established as the center of origin and diversity with abundant plant species and endemism based on its variety in geomorphology, topography and climate [35]. Furthermore, southeastern Turkey exhibits great genetic diversity of plants in the *Triticeae* family, and is supposed to be the origin of domestication for wheat and einkorn (*T. monococcum*) [36]. Among the six collection areas in this work, southeastern Turkey showed the highest genetic diversity of LMW-GS genes (35 of the total 39 variants) and genotypes (13 of the total 15 genotypes) in *T. urartu* (Figures 3b, 4a; Table 2). Almost all of the genes/variants and genotypes detected in the remaining areas were also detected in southeastern Turkey. Moreover, many variants (e.g., *TuA3-463, TuA3-498*, *TuA3-535*, *TuA3-576c*) and genotypes (U1, U3, U4, U5, U11, U12 and U14) were unique to southeastern Turkey (Figures 3b, 4a; Table 2). Even though the U8 genotype was exclusively detected in northeastern Lebanon and Syria and the U15 genotype was specifically identified in Armenia, similar genotypes were widely present in southeastern Turkey (U8 with U10 and U15 with U2 and U7) (Table 2). Considering the largest genetic diversity and typical LMW-GS genes/variants and genotypes, southeastern Turkey might be the center of origin and diversity of *T. urartu*. This conclusion was confirmed by the analysis of the loci coding storage proteins [29,37] and the assessment of AFLP markers [25].

Lebanon was supposed as a center of specific adaptation for diploid and tetraploid wheats given that some morphological characters were exclusively detected there [38]. However, fewer unique LMW-GS genes/variants and genotypes were detected in *T. urartu* accessions from northeastern Lebanon than those from southeastern Turkey (Figures 3b, 4a; Table 2). Northwestern Syria was regarded as one of the regions of richest genetic diversity of *T. urartu* based on the assessment by AFLP markers [25]. Iran is one of the primary centers of diversity for wheat and its relatives; wild wheats, in particular diploid species, are extensively distributed in its various parts [39]. However, low genetic diversity of *T. urartu* in Syria and Iran was detected due to the lack of accessions collected, and the LMW-GS genes/variants and genotypes identified in these two areas were shared by southeastern Turkey and/or northeastern Lebanon (Figures 3b, 4a; Table 2). Larger collections of *T. urartu* are needed for further analyses to draw more precise conclusions about the diversity of LMW-GS genes/variants and genotypes in Syria and Iran.

### Direct A genome donors of *T. aestivum*

Common wheat (AABBDD) is believed to be the result of spontaneous crosses between *T. dicoccoides* (A^u^A^u^BB) and *Ae. tauschii* (DD); *T. dicoccoides* (A^u^A^u^BB) was produced by the hybridization between *T. urartu* (A^u^A^u^) and the B genome ancestor which was speculated as *Ae. speltoides* (SS) [2]. Considering its wide adaptability and variation, common wheat is believed to have arisen more than once from crosses of different genotypes of its progenitor species [40,41]. The determination of the specific donors of the A genome of bread wheat would benefit not only the genetic diversity conservation of *T. urartu* but expand the genetic basis for bread wheat breeding. The dissection of the LMW-GS gene family certainly would provide some evidence about the direct donors of the A genome of common wheat.

*T. urartu* and common wheat shared two genes, *A3-391* and *A3-400* (Figure 5a). The allelic variants for each gene showed high identity (>97%), thus it was difficult to match the allelic variants between *T. urartu* and common wheat. The other two genes, *TuA3-385* and *TuA3-460* were unique to *T. urartu* (Figure 5a). The i-type genes were present as haplotypes and showed high diversity in common wheat and *T. urartu*. Except *A3-502* shared by *T. urartu* and common wheat (Figure 5b), the other i-type genes in common wheat were divided into five groups, from i_A_-1 to i_A_-5 [17], of which i_A_-3 (*A3-573*/*A3-640*) and i_A_-4 (*A3-649-1*/*A3-649-2*) contained the same number of i-type genes with *T. urartu* (Figure 5b). The *TuA3-538* genes showed close relationship with *A3-640* (i_A_-3) and *A3-649-2* (i_A_-4), and the *TuA3-576* genes showed higher identity with *A3-573* (i_A_-3) and *A3-649-1* (i_A_-4) than the other genes (Figure 5b). Thus, the characterized *T. urartu* might be the direct donor of the *Glu-A3* locus of common wheat varieties possessing i-type genes i_A_-3 and i_A_-4. Moreover, the i-type genes i_A_-3 and i_A_-4 should be the ancient genotypes because they had the same number of i-type genes with *T. urartu* and their genes closely matched those in *T. urartu* with high identity (Figure 5b) [17]. Interestingly, group i_A_-4 were only detected from landraces in the micro-collection of Chinese wheat germplasm, which also suggested that i_A_-4 might be an ancient genotype [17]. The i_A_-1 and i_A_-2 groups might also be derived from the characterized *T. urartu* because their i-type genes shared the same branch with *TuA3-576*. But i_A_-1 and i_A_-2 groups only contained one i-type genes, which shared higher identity with *TuA3-576* than *TuA3-538* (Figure 5b). Thus, in these genotypes, *TuA3-538* was lost due to a deletion and many SNP mutations of *TuA3-576* were introduced during polyploidization. The i_A_-5 was a special group of i-type genes because all three genes were substantially different from the i-type genes in *T. urartu* (Figure 5b). This group of i-type genes in common wheat might be derived from some other LMW-GS genotypes not detected in the present study, or they might have undergone many deletion and duplication processes during their evolution.

## Conclusions

In summary, this work has promoted our understanding of the composition, variation, expression and evolution of LMW-GS genes in *T. urartu*. Analysis of the geographic distribution of LMW-GS genes/variants and genotypes would facilitate the in situ conservation of the genetic diversity of *T. urartu*. These new LMW-GS genes/variants would broaden the genetic resources in wheat quality breeding and accelerate their application to improve bread-making quality in common wheat.

## Methods

### *T. urartu* accessions

The *T. urartu* accessions were obtained from the State Key Laboratory of Plant Cell and Chromosome Engineering, the Institute of Genetics and Developmental Biology, and the Chinese Academy of Sciences. This collection consisted of 157 accessions including 82 from northeastern Lebanon (Iaat, Kfardane, Talia and Baalbek), 63 from southeastern Turkey (Mardin and Urfa), five from Armenia, five from Syria (Damascus and Haseke), one from Iraq (Arbil) and one from Iran (Bakhtaran) (Additional file 1: Table S1).

### DNA isolation and polymerase chain reaction (PCR) amplification

Genomic DNA of 157 *T. urartu* accessions were extracted from young leaves of 14-day-old seedlings grown in a glasshouse using the cetyltrimethyl ammonium bromide (CTAB) method [42]. PCR was conducted using 20-μl reaction volumes consisting 1.0 U LA Taq DNA polymerase (Takara Bio, Otsu, Japan), 1 × GC buffer I (Mg^2+^, plus), 8 nM of each dNTP, 100 ng of genomic DNA and 6 pmol of each specific primer. The PCR reactions were performed using a Veriti Thermal Cycler (Applied Biosystems, Foster City, CA, USA) according to Zhang *et al*. [13].

### Detection of LMW-GS genes

LMW-GS genes were detected using the LMW-GS gene molecular marker system [13]. PCR products were purified with 3.0 M sodium acetate and 70% ethanol before adding HiDi-formamide and GeneScan 1200 LIZ size standard (Applied Biosystems, Foster City, CA, USA). DNA fragments of LMW-GS genes were separated by capillary electrophoresis using a 3730*xl* DNA Analyzer (Applied Biosystems, Foster City, CA, USA) with the default genotyping module and the G5 dye set. LMW-GS genes and allelic variants were designated in accordance with the size of their corresponding fragment lengths in the GeneMapper Software v3.7 (e.g., 385 and 397) [13].

### Cloning of LMW-GS genes

Fifty accessions were chosen to clone LMW-GS genes whose DNA fragment lengths were detected by the marker system [15]. Genes were cloned using the full length gene method and were further nominated as per the above cloning method [15]. Briefly, those sequences with high identity but a different length of repetitive domains were assigned to a single gene. Conversely, in a single gene, those sequences of conserved SNPs or different fragment lengths were considered allelic variants of the gene. Each gene was represented by the variant detected in the majority of accessions and designated as ‘representative variant DNA fragment length + gene’. Similarly, allelic variants were named, ‘DNA fragment length + allele’, and letters in alphabetical order were added to distinguish these variants with the same fragment lengths but different SNPs according to their frequencies in the *T. urartu* population. For example, considering their high identity (>99%), *TuA3-460*, *TuA3-463* and *TuA3-474* were regarded as allelic variants of the gene *TuA3-460*; for *TuA3-460* was detected in the majority of accessions (21), whereas *TuA3-463* and *TuA3-474* were only in three and four accessions, respectively (Additional file 2: Figure S8).

### Analysis of LMW-GS gene sequences

The assembly and alignment of LMW-GS gene sequences were performed with the Lasergene software (DNASTAR; http://www.dnastar.com/). Sequence alignment results were visualized with GeneDoc (http://www.nrbsc.org/gfx/genedoc/ebinet.htm). The phylogenetic trees of DNA sequences or predicted protein sequences of LMW-GS genes were constructed using the ClustalW2 (http://www.ebi.ac.uk/Tools/msa/clustalw2) and MEGA5 software [43] with the Neighbor-joining method.

### Separation and characterization of LMW-GSs

To elucidate the expression pattern of LMW-GS genes in *T. urartu*, four accessions, which in turn contained one (PI428202), two (PI428255), three (PI428270) and four (PI428335) LMW-GS genes with intact ORFs, were chosen for proteomic analysis. In each accession, glutenins were extracted from three seeds with their embryos removed [44]. Then, the prepared glutenin samples were separated by 2-DE [12], and all the spots on 2-DE gels of PI428202, PI428255 and PI428270, were digested by chymotrypsin (Sigma-Aldrich, MO, USA) and identified by LC-MS/MS [12,45]. The LC-MS/MS spectra were analyzed with Bioworks 3.1 software, using a database including protein sequences of *Triticeae* available in NCBI (before 2013-7), deduced amino acid sequences from the *T. urartu* genomic data (http://gigadb.org/dataset/100050) and protein sequences of LMW-GS genes cloned in this work. The unidentified spots were further analyzed using the MALDI-TOF/TOF mass spectrometry (AB SCIEX 5800). MS and MS/MS data were analyzed using MASCOT 2.0 search engine (Matrix Science, London, U.K.) to search against the same database of the former LC-MS/MS, with the peptide mass tolerance and the MS/MS ion tolerance of 0.2 Da and 0.5 Da, respectively. The protein scores greater than 58 were significant (p < 0.05). Considering the identical electrophoretic mobility of the above three accessions, only the spots of LMW-GSs in PI428335 were selected for mass spectra analysis. After verifying its consistency with 2-DE, SDS-PAGE was exploited to separate the LMW-GSs of every *T. urartu* accession for its high efficiency.

### Availability of supporting data

The resulting 148 LMW-GS sequences data were deposited in GenBank (http://www.ncbi.nlm.nih.gov) under the accessions of KM065455-KM065457 and KM085178-KM085322. Other supporting data, Additional file 2.pdf and Additional file 1.pdf, are included as additional files of this manuscript.

## Additional files

Additional file 1: Table S1. Collection site of the 157 *T. urartu* accessions. **Table S2**. Composition and variation of LMW-GS genes in *T. urartu* population. **Table S3.** Matching LMW-GS protein spots detected by 2-DE to the proteins predicted from the cloned active LMW-GS genes in *T. urartu* based on their Mass spectra. **Table S4.** LC-MS/MS identification of the protein spots on 2-DE gels of the glutenin fraction from *T. urartu* accessions. **Table S5.** MOLDI-TOF/TOF identification of the protein spots on 2-DE gels of the glutenin fraction from *T. urartu* accessions. **Table S6.** Nucleotide sequence identities of LMW-GS genes from *T. urartu* to the previously reported genes/allelic variants. Additional file 2: Figure S1. Sequence alignments of the *TuA3-385* gene identified in *T. urartu* and its homologs in common wheat and *Ae. tauschii*. **Figure S2.** Sequence alignments of the *TuA3-391* gene identified in *T. urartu*. **Figure S3.** Sequence alignments of the *TuA3-397* and *TuA3-400* genes identified in *T. urartu*. **Figure S4.** Sequence alignments of the *TuA3-502* gene identified in *T. urartu*. **Figure S5.** Sequence alignments of the *TuA3-538* gene identified in *T. urartu*. **Figure S6.** Sequence alignments of the *TuA3-576* gene identified in *T. urartu*. **Figure S7.** Protein sequence alignments of variants of *TuA3-460* gene with s- and m-type genes characterized previously at the *Glu-B3* and *Glu-D3* loci in common wheat and m-type genes in *T. urartu.* **Figure S8**. Sequence alignments of the *TuA3-460* gene identified in *T. urartu*. **Figure S9.** Sequence alignments of the deduced proteins of all active LMW-GS genes in *T. urartu*.

## Abbreviations

2-DE: Two-dimensional electrophoresis
CTAB: Cetyltrimethyl ammonium bromide
HMW-GS: High-molecular-weight glutenin subunit
LC-MS/MS: Liquid chromatography tandem mass spectrometry
LMW-GS: Low-molecular-weight glutenin subunit
MALDI-TOF/TOF-MS: Matrix assisted laser desorption/ionization time of flight tandem mass spectrometry
MCC: Micro-core collections
ORF: Open reading frame
PCR: Polymerase chain reaction
pI: Isoelectric point
SDS-PAGE: Sodium dodecyl sulphate polyacrylamide gel electrophoresis
