# Supplementary material for: Composition, variation, expression and evolution of low-molecular-weight glutenin subunit genes in Triticum urartu
Source: BMC Plant Biol. 2015 Feb 28;15:68. doi: 10.1186/s12870-014-0322-3 (PMC4364320; doi:10.1186/s12870-014-0322-3)
Supplement: Additional file 1: Table S1. — Collection site of the 157 T. urartu accessions. Table S2. Composition and variation of LMW-GS genes in T. urartu population. Table S3. Matching LMW-GS protein spots detected by 2-DE to the proteins predicted from the cloned active LMW-GS genes in T. urartu based on their Mass spectra. Table S4. LC-MS/MS identification of the protein spots on 2-DE gels of the glutenin fraction from T. urartu accessions. Table S5. MOLDI-TOF/TOF identification of the protein spots on 2-DE gels of the glutenin fraction from T. urartu accessions. Table S6. Nucleotide sequence identities of LMW-GS genes from T. urartu to the previously reported genes/allelic variants. [file 12870_2014_322_MOESM1_ESM.pdf]

ADDITIONAL FILE 2

Composition, variation, expression and evolution of low-molecular-weight glutenin subunit gene family in *Triticum urartu*

Guangbin Luo[1, 2], Xiaofei Zhang[1,*], Yanlin Zhang[3], Wenlong Yang[1], Yiwen Li[1], Jiazhu Sun[1], Kehui Zhan[3], Aimin Zhang[1, 3, 4], Dongcheng Liu[1, 4]

Table S1. Collection site of the 157 *T. urartu* accessions.

Table S2. Composition and variation of LMW-GS genes in *T. urartu* population.

Table S3. Matching LMW-GS protein spots detected by 2-DE to the proteins predicted from the cloned active LMW-GS genes in *T. urartu* based on their Mass spectra.

Table S4. LC-MS/MS identification of the protein spots on 2-DE gels of the glutenin fraction from *T. urartu* accessions.

Table S5. MOLDI-TOF/TOF identification of the protein spots on 2-DE gels of the glutenin fraction from *T. urartu* accessions.

Table S6. Nucleotide sequence identities of LMW-GS genes from *T. urartu* to the previously reported genes/allelic variants.

Table S1. Collection site of the 157 *T. urartu* accessions. *Accessions chosen for gene cloning.

Genotype	Accession	Collection site	Altitude (m)
U1	PI428318*	Urfa, Turkey	NA
U2	CItr17667	Lebanon	NA
	PI428184*	Mardin, Turkey	600
	PI428189	Mardin, Turkey	600
	PI428190*	Mardin, Turkey	600
	PI428195	Mardin, Turkey	600
	PI428202*	Mardin, Turkey	625
	PI428207	Mardin, Turkey	625
	PI428216	Mardin, Turkey	625
	PI428230	Urfa, Turkey	600
	PI428232	Urfa, Turkey	600
	PI428234	Urfa, Turkey	600
	PI428237	Urfa, Turkey	600
	PI428238	Urfa, Turkey	600
	PI428239	Urfa, Turkey	600
	PI428240	Urfa, Turkey	600
	PI428242	Urfa, Turkey	600
	PI428244	Urfa, Turkey	600
	PI428245	Urfa, Turkey	600
	PI428246	Urfa, Turkey	600
	PI428248	Urfa, Turkey	600
	PI428253	Arbil,Iraq	1100
	PI428265*	Baalbek, Lebanon	1000
	PI428295*	Baalbek, Lebanon	1000
	PI428301	Baalbek, Lebanon	1000
	PI428303	Baalbek, Lebanon	1000
	PI428316	Bakhtaran,Iran	1650
	PI428321	Talia, Lebanon	1030
	PI428326	Kfardane, Lebanon	1080
	PI428332	Kfardane, Lebanon	1080
	PI487268	Haseke,Syria	NA
	PI538728	Urfa, Turkey	600
	PI538729	Urfa, Turkey	600
	PI538730	Urfa, Turkey	600
	PI538733	Urfa, Turkey	600
	PI538747	Talia, Lebanon	1030
U3	PI428196*	Mardin, Turkey	600
	PI428203	Mardin, Turkey	625

	PI428209*	Mardin, Turkey	625
	PI428215	Mardin, Turkey	625
	PI428219*	Mardin, Turkey	625
U4	PI428188*	Mardin, Turkey	600
	PI428197*	Mardin, Turkey	600
	PI428208*	Mardin, Turkey	625
	PI428212*	Mardin, Turkey	625
	PI428213	Mardin, Turkey	625
	PI428225	Mardin, Turkey	625
	PI428231	Urfa, Turkey	600
U5	PI428187*	Mardin, Turkey	600
	PI428193	Mardin, Turkey	600
	PI428194*	Mardin, Turkey	600
	PI428199	Mardin, Turkey	600
	PI428200*	Mardin, Turkey	600
	PI428201*	Mardin, Turkey	600
	PI428206	Mardin, Turkey	625
	PI428211*	Mardin, Turkey	625
	PI428218	Mardin, Turkey	625
U6	CItr17666	Lebanon	NA
	PI428235*	Urfa, Turkey	600
	PI428261*	Baalbek, Lebanon	1000
	PI428262	Baalbek, Lebanon	1000
	PI428263	Baalbek, Lebanon	1000
	PI428264	Baalbek, Lebanon	1000
	PI428271	Baalbek, Lebanon	1000
	PI428273	Baalbek, Lebanon	1000
	PI428275	Baalbek, Lebanon	1000
	PI428278*	Baalbek, Lebanon	1000
	PI428279	Baalbek, Lebanon	1000
	PI428281	Baalbek, Lebanon	1000
	PI428282	Baalbek, Lebanon	1000
	PI428285	Baalbek, Lebanon	1000
	PI428286*	Baalbek, Lebanon	1000
	PI428287*	Baalbek, Lebanon	1000
	PI428289	Baalbek, Lebanon	1000
	PI428291	Baalbek, Lebanon	1000
	PI428292	Baalbek, Lebanon	1000
	PI428294	Baalbek, Lebanon	1000
	PI428298	Baalbek, Lebanon	1000
	PI428299	Baalbek, Lebanon	1000

	PI428300*	Baalbek, Lebanon	1000
	PI428302	Baalbek, Lebanon	1000
	PI428304	Baalbek, Lebanon	1000
	PI428310	Baalbek, Lebanon	1000
	PI428312*	Baalbek, Lebanon	1000
	PI428313	Baalbek, Lebanon	1000
	PI428314	Baalbek, Lebanon	1000
	PI428330	Kfardane, Lebanon	1080
	PI428333*	Iaat, Lebanon	1050
	PI428334	Iaat, Lebanon	1050
	PI487266	Haseke,Syria	520
	PI487270	Damascus,Syria	NA
	PI538734*	Baalbek, Lebanon	1000
	PI538737	Baalbek, Lebanon	1000
	PI538738	Baalbek, Lebanon	1000
	PI538739	Baalbek, Lebanon	1000
	PI538743	Baalbek, Lebanon	1000
U7	PI428191*	Mardin, Turkey	600
	PI428269*	Baalbek, Lebanon	1000
U8	PI428266	Baalbek, Lebanon	1000
	PI428272	Baalbek, Lebanon	1000
	PI428290*	Baalbek, Lebanon	1000
	PI428293	Baalbek, Lebanon	1000
	PI428305	Baalbek, Lebanon	1000
	PI428320	Talia, Lebanon	1030
	PI428322*	Talia, Lebanon	1030
	PI428324	Kfardane, Lebanon	1080
	PI428327	Kfardane, Lebanon	1080
	PI428335*	Iaat, Lebanon	1050
	PI428336	Iaat, Lebanon	1050
	PI428338	Iaat, Lebanon	1050
	PI487265	Haseke,Syria	470
	PI538736	Baalbek, Lebanon	1000
	PI538742	Baalbek, Lebanon	1000
	PI538749	Kfardane, Lebanon	1050
U9	PI428224*	Mardin, Turkey	625
	PI428228	Mardin, Turkey	NA
	PI428229*	Mardin, Turkey	NA
	PI428255*	Mardin, Turkey	NA
	PI428267	Baalbek, Lebanon	1000
	PI487267	Haseke,Syria	NA

U10	CItr17664	Lebanon	NA
	DV2138*	Urfa, Turkey	NA
	PI428258	Erevan,Armenia	NA
	PI428260	Baalbek, Lebanon	1000
	PI428270*	Baalbek, Lebanon	1000
	PI428276	Baalbek, Lebanon	1000
	PI428283	Baalbek, Lebanon	1000
	PI428288	Baalbek, Lebanon	1000
	PI428296	Baalbek, Lebanon	1000
	PI428297	Baalbek, Lebanon	1000
	PI428306	Baalbek, Lebanon	1000
	PI428309	Baalbek, Lebanon	1000
	PI428311*	Baalbek, Lebanon	1000
	PI428323	Kfardane, Lebanon	1080
	PI428325*	Kfardane, Lebanon	1080
	PI428328	Kfardane, Lebanon	1080
	PI428329*	Kfardane, Lebanon	1080
	PI428340	Iaat, Lebanon	1050
	PI538735	Baalbek, Lebanon	1000
	PI538740	Baalbek, Lebanon	1000
	PI538751	Iaat, Lebanon	1050
U11	PI428192*	Mardin, Turkey	600
	PI428204*	Mardin, Turkey	625
U12	PI428220*	Urfa, Turkey	670
U13	PI428250*	Urfa, Turkey	500
	PI428251*	Urfa, Turkey	500
	PI428274*	Baalbek, Lebanon	1000
	PI554599	Urfa, Turkey	NA
U14	PI428186*	Mardin, Turkey	600
	PI428198*	Mardin, Turkey	600
	PI428214	Mardin, Turkey	625
	PI428222	Urfa, Turkey	670
	PI554597*	Urfa, Turkey	600
U15	CItr17668*	Armenia	NA
	PI428180	Armenia	NA
	PI428181	Armenia	NA
	PI428182	Armenia	NA

Table S2. Composition and variation of LMW-GS genes in *T. urartu* population.

Type	Gene	Allelic variant	Accession Number
**m-type**	**TuA3-385**	TuA3-385a	110
		TuA3-385b	43
	**TuA3-391**	TuA3-373a	25
		TuA3-391	40
		TuA3-392	92
	**TuA3-397**	TuA3-397a	137
		TuA3-397b	20
	**TuA3-400**	TuA3-400	4
		TuA3-402	5
**S-type**	**TuA3-460**	TuA3-460	21
		TuA3-463	3
		TuA3-474	4
**i-type**	**TuA3-502**	TuA3-495	1
		TuA3-498	5
		TuA3-502a	76
		TuA3-502b	35
		TuA3-502c	14
		TuA3-502d	2
		TuA3-520	14
		TuA3-590	4
		TuA3-593	6
	**TuA3-538**	TuA3-535	5
		TuA3-538a	53
		TuA3-538b	46
		TuA3-538c	35
		TuA3-538d	11
		TuA3-538e	6
		TuA3-657	1
	**TuA3-576**	TuA3-406	35
		TuA3-555	2
		TuA3-576a	39
		TuA3-576b	15
		TuA3-576c	14
		TuA3-576d	5
		TuA3-576e	4
		TuA3-579a	21
		TuA3-579b	16
		TuA3-597	5
		TuA3-669	1
**Total**			157

Table S3. Matching LMW-GS protein spots detected by 2-DE to the proteins predicted from the cloned active LMW-GS genes in *T. urartu* based on their Mass spectra. *Methionine residue with oxidation modification.

Spot	Mass spectrum	MH^+	Charge	XC	Matching gene	Predicted LMW-GS protein sequence	Coverage
PI428202-U2_1	F.SQQQQIPVIHPSVL.Q	1573.87	2	3.15	TuA3-538c	ISQQQQAPPFSQQQQPPFSQQQQPPFSQQQQSPFSQQQQQPPFA	14.46%
	I.PVIHPSVL.Q	861.52	1	1.90		QQQQPPFSQQPPISQQQQPPFSQQQQPQFSQQQQPPYSQQQQPP	
	L.GQCVSQPQQELQQQL.G	1770.84	2	3.17		YSQQQQPPFSQQQQPPFSQQQQQPPFTQQQQPPFSQQPPISQQQ	
	Q.PPFSQQQQIPVIHPSVL.Q	1915.04	2	3.00		QPPFSQQQQPPFSQQQQIPVIHPSVLQQLNPCKLFLEQQCIPVA	
	Q.PPFSQQQQPPFSQQQQIPVIHPSVL.Q	2855.48	2	3.25		MQQCLARSQMLEQSICHVMQQQCCQQLRQIPEQSRHESIRAIV	
	Y.QQQQPQQLGQCVSQPQQELQQQL.G	2749.33	3	4.01		YSIILQQQQQQQQQQQQQQQQGQSIIQYQQQQPQQLGQCVSQ	
						PQQELQQQLGQQPQQQQLAHGTFLQPHQIAQLEVMTSIALRN	
						LPTMCSVNVPLYETTTSVPLGVGIGVGVY	
PI428202-U2_2	F.LQPHQIAQLEVM*.T	1422.75	1	2.10	TuA3-538c	ISQQQQAPPFSQQQQPPFSQQQQPPFSQQQQSPFSQQQQQPPFA	17.17%
	F.SQQQQIPVIHPSVL.Q	1573.87	2	2.67		QQQQPPFSQQPPISQQQQPPFSQQQQPQFSQQQQPPYSQQQQPP	
	F.SQQQQPPFSQQQQIPVIHPSVL.Q	2514.31	2	3.32		YSQQQQPPFSQQQQPPFSQQQQQPPFTQQQQPPFSQQPPISQQQ	
	F.SQQQQSPF.S	949.44	1	1.53		QPPFSQQQQPPFSQQQQIPVIHPSVLQQLNPCKLFLEQQCIPVA	
	H.ESIRAIVY.S	950.53	1	1.68		MQQCLARSQMLEQSICHVMQQQCCQQLRQIPEQSRHESIRAIV	
	I.PVIHPSVL.Q	861.52	1	1.68		YSIILQQQQQQQQQQQQQQQQGQSIIQYQQQQPQQLGQCVSQ	
	L.RNLPTMC.S	891.45	1	2.01		PQQELQQQLGQQPQQQQLAHGTFLQPHQIAQLEVMTSIALRN	
	Q.PPFSQQQQIPVIHPSVL.Q	1915.04	2	3.14		LPTMCSVNVPLYETTTSVPLGVGIGVGVY	
PI429270-U10_1	F.LQPHQIAQLEVM.T	1406.75	2	2.93	TuA3-538b	ISQQQQAPPFSQQQQPPFSQQQQPPFLQQQQSPFSQQQQQPPFA	37.16%
	F.SQQQQIPVIHPSVL.Q	1573.87	2	3.50		QQQQPPFSQQPPISQQQQPPFSQQQQPQFSQQQHPPYSQQQQPP	
	F.SQQQQPPFLQQQQSPF.S	1915.93	2	3.31		YSQQQQPPFSQQQQPPFSQQQQQPPFTQQQQPPFSQQPPISQQQ	

	F.SQQQQPQFSQQQHPPY.S	1955.90	2	3.24		QPPFSQQQQPPFSQQQQIPVIHPSVLQQLNPCKVFLQQQCIPVA	
	F.TQQQQPPFSQQPPISQ.Q	1838.90	2	2.40		MQRCLARSQMLQQSICHVMQQQCCQQLRQIPEQSRHESIRAIV	
	I.PVIHPSVL.Q	861.52	1	1.83		YSIILQQQQQQQQQQQQQQQGQSIIQYQQQRPQQLGQCVSQP	
	L.GQCVSQPQQELQQQL.G	1770.88	2	4.13		QQELQQQLGQQPQQQQLAHGTFLQPHQIAQLEVMTSIALRNL	
	L.GVGIGVG.A	558.33	1	1.54		PTMCSVNVPLYETTTSVPLGVGIGVGAY	
	Q.CVSQPQQELQQQL.G	1585.80	2	2.45			
	Q.IPVIHPSVL.Q	974.60	1	1.81			
	Q.PHQIAQL.E	806.45	1	1.64			
	Q.PPFLQQQQSPF.S	1316.66	1	1.98			
	Q.PPFSQQQQIPVIHPSVL.Q	1915.04	2	3.08			
	Q.PPFTQQQQPPF.S	1314.65	1	2.07			
	Q.QQQQQQQQQQQQQGQSIIQYQQ.Q	2729.30	2	2.62			
	Q.QQQQQQQQQQQQQQGQSIIQYQ.Q	2729.30	2	2.75			
PI429270-U10_2	F.SQQQQIPVIHPSVL.Q	1573.87	2	3.34	TuA3-579a	ISQQQQPPPPFSEQQQPPFSQQQQPPFSQQQQSPFSQQQQQPPFS	16.47%
	F.SQQQRPPFSQQQQIPVIHPSVL.Q	2542.35	2	2.28		QQQQPPFSQQPPISQQQQPPFSQQQQPQFSQQQQPPYSQQQQPP	
	H.ESIRAIIY.S	964.55	1	1.67		YSQQQQPPFSQQQQPPFSQQQQQPPISQQQQQQQQQQQPFTQQ	
	H.VMQQQCCQ.Q	1081.42	2	2.87		QQPPFSQQPPISQQQQPPFSQQQRPPFSQQQQIPVIHPSVLQQLN	
	I.PVIHPSVL.Q	861.52	1	1.65		PCKVFLQQQCIPVAMQRCLARSQMLQQSICHVMQQQCCQQL	
	M.LQQSICH.V	885.43	1	1.64		RQIPEQSRHESIRAIIYSIILQQQQQQQQQQQQQQQQQGQSIIQY	
	Q.IPVIHPSVL.Q	974.60	1	1.76		HQQQPQQLGQCVSQPLQQLQQQLGQQPQQQQLAHQIAQLEV	
	Y.HQQQPQQL.G	1006.51	1	1.98		MTSIALRTLPTMCSVNVPLYETTTSVPLGVGIGVGVY	
	Y.HQQQPQQLGQ.C	1191.59	2	3.00			
PI429270-U10_3	I.PVIHPSVL.Q	861.52	1	1.69	TuA3-502a	ISQQQQQQPFPQQQQPPFSQQQEPTYSQQQQPPFSQQQQSPFSQ	27.41%
	L.GQCVSQPQQQSQQQL.G	1743.84	2	3.82		QQQQQPPFLQQHQPPFSQQPPISQQQQPPFSQQQQPPFSQQQQP	

	Q.CVSQPQQQSQQQL.G	1558.76	2	2.31		PFSQQQQQQQPPFSQQQQPPFSQQPPISQQQQPPFSQQQQPPFS	
	Q.PPFSQQ.Q	703.34	1	1.56		QQQQIPVIHPSVLQQLNPCKVFLQQQCIPVAMQRCLARSQMLQ	
	Q.PPFSQQQ.Q	831.40	1	1.68		QRICHVMQQQCCQQLRQIPGQSRHESIRAIIYSIILQQQQQQQ	
	Q.QLGQQPQQQQL.A	1295.67	1	1.71		QQQQQQQQQVQSIIQAQQQQPQQLGQCVSQPQQQSQQQLG	
	Q.QQLGQQPQQQQL.A	1423.73	1	1.82		QQPQQQQLAQGTFLQPHQIAQFEVMTSIALRTLPTMCNVNVPL	
						YGTTTSAPFGVGTGVGAY	
PI428335-U8_1	F.LQPHQIAQLEVM.T	1406.75	2	3.40	TuA3-538b	ISQQQQAPPFSQQQQPPFSQQQQPPFLQQQQSPFSQQQQQPPFA	31.02%
	F.LQQQCIPVAM*QR.C	1487.78	2	2.65		QQQQPPFSQQPPISQQQQPPFSQQQQPQFSQQQHPPYSQQQQPP	
	F.SQQQQIPVIHPSVL.Q	1573.87	2	3.36		YSQQQQPPFSQQQQPPFSQQQQQPPFTQQQQPPFSQQPPISQQQ	
	H.ESIRAIVY.S	950.53	1	1.58		QPPFSQQQQPPFSQQQQIPVIHPSVLQQLNPCKVFLQQQCIPVA	
	I.PVIHPSVL.Q	861.52	1	1.92		MQRCLARSQMLQQSICHVMQQQCCQQLRQIPEQSRHESIRAIV	
	L.GQCVSQPQQELQQQL.G	1770.88	2	2.48		YSIILQQQQQQQQQQQQQQGQSIIQYQQQRPQQLGQCVSQP	
	L.QQLNPCKVF.L	1133.61	2	2.97		QQELQQQLGQQPQQQQLAHGTFLQPHQIAQLEVMTSIALRNL	
	Q.CVSQPQQELQQQL.G	1585.80	2	2.81		PTMCSVNVPLYETTTSVPLGVGIGVGAY	
	Q.PPFSQQQQIPVIHPSVL.Q	1915.04	2	3.09			
	Q.PPFSQQQQPPFSQQQQIPVIHPSVL.Q	2855.48	2	2.19			
	Q.QQQQQQQQQQQQQQGQSIIQYQ.Q	2729.30	3	3.40			
PI428335-U8_2	F.SQQQQIPVIHPSVL.Q	1573.87	2	2.58	TuA3-579b	ISQQQQPPPFSEQQQPPFSQQQQPPFSQQQQSPFSQQQQQPPFS	23.08%
	F.SQQQQPPFSQQQQSPF.S	1889.88	2	3.07		QQQQPPFSQQPPISQQQQPPFSQQQQPQFSQQQQPPYSQQQQPP	
	F.SQQQRPPFSQQQQIPVIHPSVL.Q	2542.35	3	4.31		YSQQQQPPFSQQQQPPFSQQQQQPPISQQQQQQQQQQPFTQQ	
	H.ESIRAIIY.S	964.55	1	1.54		QQPPFSQQPPISQQQQPPFSQQQRPPFSQQQQIPVIHPSVLQQLN	
	I.PVIHPSVL.Q	861.52	1	1.60		PCKVFLQQQCIPVAMQRCLARSQMLQQSICHVMQQQCCQQL	
	L.AHQIAQLEVM.T	1139.59	2	2.45		RQIPEQSRHESIRAIIYSIILQQQQQQQQQQQQQQGQSIIQYQQ	
	L.QQLNPCKVF.L	1133.61	2	2.64		QQPQQLGQCVSQPLQQLQQQLGQQPQQQQLAHQIAQLEVMT	

	L.RTLPTMCSVNVPL.Y	1487.80	2	2.15		SIALRTLPTMCSVNVPLYETTTSVPLGVGIGVGVY	
	Q.QRPPFSQQQQIPVIHPSVL.Q	2199.20	3	2.71			
PI428335-U8_3	F.LQPHQIAQF.E	1081.58	2	2.97	TuA3-502a	ISQQQQQQPFPQQQQPPFSQQQEPTYSQQQQPPFSQQQQSPFSQ	31.06%
	F.LQPHQIAQFEVM.T	1440.73	2	2.28		QQQQQPPFLQQHQPPFSQQPPISQQQQPPFSQQQQPPFSQQQQP	
	F.SQQQQIPVIHPSVL.Q	1573.87	2	3.68		PFSQQQQQQQPPFSQQQQPPFSQQPPISQQQQPPFSQQQQPPFS	
	F.SQQQQPPFSQQQQIPVIHPSVL.Q	2514.31	2	2.61		QQQQIPVIHPSVLQQLNPCKVFLQQQCIPVAMQRCLARSQMLQ	
	F.SQQQQSPF.S	949.44	1	1.68		QRICHVMQQQCCQQLRQIPGQSRHESIRAIIYSIILQQQQQQQ	
	I.PVIHPSVL.Q	861.52	1	1.89		QQQQQQQQQQVQSIIQAQQQQPQQLGQCVSQPQQQSQQQLG	
	L.QQLNPCKVF.L	1133.61	2	2.53		QQPQQQQLAQGTFLQPHQIAQFEVMTSIALRTLPTMCNVNVPL	
	Q.PPFSQQ.Q	703.34	1	1.51		YGTTTSAPFGVGTGVGAY	
	Q.PPFSQQQQIPVIHPSVL.Q	1915.04	2	2.71			
	Q.PPFSQQQQPPFSQQQQIPVIHPSVL.Q	2855.48	2	2.60			
	Q.PPFSQQQQSPF.S	1290.61	1	2			
	Q.SIIQAQQQQPQQL.G	1509.80	2	2.95			
PI428335-U8_4	C.IPGLERPW.Q	967.54	1	1.84	TuA3-397b	MDTSCIPGLERPWQQQPLPPQQTFPQQPPFSQQQQQQQPFPQQ	32.03%
	F.LQPHQIAHLEVM.T	1415.75	1	2.42		PSFSQQQPPFSQQQPILPQGPPFSQQTQPVLPQQSPFSQQQQLIL	
	F.VQAQQQQPQQSGQGVSQSQQQSQQ	2881.38	3	3.45		PPQQQQQLPQQQISIVQPSVLQQLNPCKVFLQQQCSPVAMPQR	
	QL.G					LARSQMWQQSSCHVMQQQCCQQLSQIPEQSRYDAIRAITYSII	
	I.PGLERPW.Q	854.45	1	1.97		LQEQQGFVQAQQQQPQQSGQGVSQSQQQSQQQLGQCSFQQP	
	L.RTLPTMCSVNVPL.Y	1487.80	1	2.02		QQQLGQQPQQQQVQQGTFLQPHQIAHLEVMTSIALRTLPTMC	
	L.RTLPTMCSVNVPLY.S	1650.87	2	2.76		SVNVPLYSSTTSVPFGVGIGVGAY	
	L.RTLPTMCSVNVPLYS.S	1737.90	2	3.10			
	Q.ISIVQPSVL.Q	955.58	1	1.61			
	Q.QQQQPFPQQPSF.S	1459.70	2	2.39			

	Y.DAIRAITY.S	922.50	1	1.78			
PI428335-U8_5	I.ILQEQQGF.V	962.49	1	1.70	TuA3-397b	MDTSCIPGLERPWQQQPLPPQQTFPQQPPFSQQQQQQQPFPQQ	14.60%
	I.LQEQQGF.V	849.41	1	1.70		PSFSQQQPPFSQQQPILPQGPPFSQQTQPVLPQQSPFSQQQQLIL	
	I.PGLERPW.Q	854.45	1	1.99		PPQQQQQLPQQQISIVQPSVLQQLNPCKVFLQQQCSPVAMPQR	
	I.SIVQPSVL.Q	842.50	1	1.58		LARSQMWQQSSCHVMQQQCCQQLSQIPEQSRYDAIRAITYSII	
	Q.ISIVQPSVL.Q	955.58	1	2.00		LQEQQGFVQAQQQQPQQSGQGVSQSQQQSQQQLGQCSFQQP	
	Q.TQPVLPQQSPF.S	1241.65	1	1.57		QQQLGQQPQQQQVQQGTFLQPHQIAHLEVMTSIALRTLPTMC	
	Y.SIILQEQQGF.V	1162.61	2	3.82		SVNVPLYSSTTSVPFGVGIGVGAY	
	L.PQQQISIVQPSVL.Q	1436.81	1	1.71			
PI428255-U9_1	C.VSQPQQELQ.Q	1056.53	2	2.82	TuA3-538e	ISQQQQPPPFSQQQQPPFSQQQQPPFSQQQQSPFSQQQQQPPFS	44.31%
	F.LQPHQIAQLEVM.T	1406.75	2	2.79		QQQQPPFSQQPPISQQQQPPFSQQQQPQFSQQQQPPYSQQQQPP	
	F.SQQQQPPFSQQQQPPFSQQQQSPF.S	2830.32	2	2.07		YSQQQQPPFSQQQQPPFSQQQQQPPISQQQQPPFSQQPPISQQQ	
	I.PVIHPSVL.Q	861.52	1	1.72		QPPFSQQQQPPFSQQQQIPVIHPSVLQQLNPCKVFLQQQCIPVA	
	I.SQQQQPPFSQQ.Q	1302.61	1	1.86		MQRCLARSQMLQQSICHVMQQQCCQQLRQIPEQSRHESIRAIV	
	L.GQCVSQPQQEL.Q	1273.62	1	2.37		YSIILQQQQQQQQQQQQQQQQQQGRSIIQYQQQQPQQLGQCV	
	L.QQLNPCKVF.L	1133.61	2	2.45		SQPQQELQQQLGQQPQQQQLAHGTFLQPHQIAQLEVMTSIAL	
	L.QQQCIPVAM*QR.C	1374.70	2	2.27		RNLPTMCSVNVPLYETTTSVPLGVGIGVGVY	
	L.RNLPTM.C	731.39	1	1.60			
	L.RNLPTMC.S	891.45	1	1.89			
	Q.PPFSQQ.Q	703.34	1	1.58			
	Q.PPFSQQQ.Q	831.40	1	1.61			
	Q.PPFSQQQQSPF.S	1290.61	1	1.58			
	Q.PPPFSQQ.Q	800.39	1	2.17			
	C.VSQPQQELQ.Q	1056.53	2	2.52			

	F.LQPHQIAQLEVM.T	1406.75	2	2.20			
	I.PVIHPSVL.Q	861.52	1	1.55			
	I.SQQQQPPFSQQ.Q	1302.61	1	2.06			
	L.GQCVSQPQQEL.Q	1273.62	1	2.23			
	L.GVGIGVGV.Y	657.39	1	1.58			
	L.GVGIGVGVY.-	820.46	1	1.91			
	L.RNLPTMC.S	891.45	1	1.80			
	Q.PPFSQQ.Q	703.34	1	1.57			
PI428255-U9_2	-.GQQPQQQQL.-	1054.53	2	3.30	TuA3-576b	ISQQQQPPPFSEQQQPPFSQQQQPPFSQQQQSPFSQQQQQPPFS	37.24%
	-.QQLNPCKVF.-	1133.58	2	2.44		QQQQPPFSQQPPISQQQQPPFSQQQQPQFSQQQQPPYSQQQQPP	
	-.QQQLGQQPQQQQL.-	1551.79	2	2.68		YSQQQQPPFSQQQQPPFSQQQQPPISQQQQQQQQQQQPFTQQQ	
	-.QQQQPQQL.-	997.51	2	2.04		QPPFSQQPPISQQQQPPFSQQQRPPFSQQQQIPVIHPSVLQQLNP	
	-.SEQQQPPF.-	960.44	1	2.07		CKVFLQQQCIPVAMQRCLARSQMLQQSICHVMQQQCCQQLR	
	-.SQQQQIPVIHPSVL.-	1573.87	2	3.20		QIPEQSRHESIRAIIYSIILQQQQQQQQQQQGQSIIQYQQQQPQQ	
	-.SQQQQPPF.-	959.46	1	1.78		LGQCVSQPLQQLQQQLGQQPQQQQLAHQIAQLEVMTSIALRT	
	-.SQQQQPPY.-	975.45	1	1.51		LPTMCSVNVPLYETTTSVPLGVGIGVGVY	
	-.SQQQQSPF.-	949.44	1	1.69			

Table S4. LC-MS/MS identification of the protein spots on 2-DE gels of the glutenin fraction from *T. urartu* accessions. *Methionine residue with oxidation modification.

Spot	Protein	Mass spectrum	MH^+	Charge	XC
PI428270-U10_18	Alpha gliadin	Q.EILQQI.L	743.43	1	1.58
		Q.QEILQQI.L	871.49	1	2.02
PI428270-U10_19	Alpha gliadin	Q.EQILQQI.L	871.49	1	1.62
PI428270-U10_20	Globulin	C.RIEPQECSVF.S	1264.63	1	2.09
		K.VRLPTAC.R	816.47	1	1.79
		L.FQAQW.G	679.32	1	1.51
		L.TGRAGEGAVGVPL.F	1183.64	1	1.63
		Q.ILEQQL.T	743.43	1	1.69
		R.QILEQQL.T	871.49	1	1.71
PI428270-U10_21	Globulin	C.RIEPQECSVF.S	1264.63	1	2.01
		L.TGRAGEGAVGVPL.F	1183.64	1	2.03
PI428270-U10_23	Alpha gliadin	A.VRVPVPQLQPQNPSQQEPQEQVPL.V	2735.45	2	2.58
		F.RPQQPYPQPQPQY.S	1626.80	2	3.05
		I.PCMDVVLQQH.N	1226.60	1	2.14
		I.PCMDVVLQQHN.I	1340.64	1	1.82
		L.PQFEEIRNL.A	1145.60	1	1.71
		Q.QILQQIL.Q	855.53	1	1.86
PI428270-U10_24	Gamma-gliadin	I.IMQQEQQEQLQ.S	1402.66	2	2.31
		S.IIM*QQEQQEQL.Q	1403.69	2	2.31
PI428270-U10_25	Gamma-gliadin	L.GTLPTMCN.V	893.42	1	1.58
		F.ASIVADIGGQ.-	930.49	1	2.14
		Q.GIQIMRPL.F	927.55	1	1.69
PI428202-U2_3	Alpha gliadin	F.RPEQPYPQPQPQ.Y	1464.72	2	2.07
		F.RPEQPYPQPQPQY.S	1627.79	2	2.38
PI428202-U2_6	Alpha gliadin	F.RPQQPYPQPQPQ.Y	1463.74	2	2.25
		Q.KQQQQPSSQVSF.Q	1391.69	2	3.28
PI428202-U2_7	Alpha gliadin	L.HQQQQKQQQPSSQVSF.Q	1912.93	2	2.57
		Q.QKQQQPSSQVSF.Q	1391.69	2	3.57
		H.QQQQKQQQPSSQVSF.Q	1775.87	2	2.21
PI428202-U2_8	Alpha gliadin	L.HQQQKQQQQPSSQVSF.Q	1912.93	2	2.23
		A.VRVPVPQ.L	794.49	1	1.96
		Q.KQQQQPSSQVSF.Q	1391.69	2	2.03
		F.RPQQPYPQPQPQY.S	1626.80	2	3.15
		L.PQFEEIRNL.A	1145.60	1	1.71
		Y.IPPYCTIAPF.G	1178.62	1	1.55
		L.PQFEEIRNL.A	1145.60	1	1.67
PI428202-U2_9	Alpha gliadin	L.HQQQKQQQQPSSQVSF.Q	1912.93	2	2.58
		A.VRVPVPQ.L	794.49	1	1.60

		Q.KQQQQPSSQVSF.Q	1391.69	2	2.51
		A.VRVPVPQLQPQNPSQQEPQEQVPL.V	2735.45	2	2.37
		Q.QILQQIL.Q	855.53	1	1.64
		L.PQFEEIRNL.A	1145.60	1	1.65
		L.QQQLIPCMDVVLQQH.N	1836.94	2	2.13
		L.QQQLIPCMDVVL.Q	1443.76	1	1.51
PI428202-U2_10	Gamma-gliadin	I.QAQQPAQL.E	883.46	1	1.63
PI428202-U2_12	Alpha gliadin	F.RPQQPYPQPQPQ.Y	1463.74	2	2.00
		F.RPQQPYPQPQPQY.S	1626.80	2	2.39
		Q.KQQQQPSSQVSF.Q	1391.69	2	2.26
		Y.LQLQPFPKPQQPY.S	1583.86	2	2.60
PI428202-U2_13	Avenin-like protein	Q.AQLLEQM*.Q	848.42	1	1.91
PI428202-U2_14	Avenin-like protein	C.QQQQQLGQQQQQQLQEQLAPCKTF.L	2913.46	3	3.05
		Q.AQLLEQM*.Q	848.42	1	2.00
PI428202-U2_15	Avenin-like protein	Q.AQLLEQM*.Q	848.42	1	1.93
PI428255-U9_3	Alpha gliadin	F.RPQQPYPQPQPQY.S	1626.80	2	2.18
		Q.AIHNVVHAIIL.H	1199.73	2	2.07
		Q.QLPQFEEIRNL.A	1386.74	2	2.21
		L.QQQLIPCMDVVLQQH.N	1836.94	2	2.94
PI428255-U9_4	Alpha gliadin	F.RPQQPYPQPQPQ.Y	1463.74	2	3.03
		Q.QKQQQQPSSQVSF.Q	1519.75	2	2.23
		A.VRVPVPQ.L	794.49	1	1.99
		K.QQQQPSSQVSF.Q	1263.60	2	2.47
		F.RPQQPYPQPQPQY.S	1626.80	2	2.20
		A.VRVPVPQLQPQNPSQQEPQ.E	2169.14	2	2.29
		Q.QLPQFEEIRNL.A	1386.74	2	2.12
		L.QQQLIPCMDVVLQQH.N	1836.94	2	2.46
		L.PQFEEIRNL.A	1145.60	1	1.55
PI428255-U9_5	Alpha gliadin	A.VRVPVPQ.L	794.49	1	1.88
		Q.QLPQFEEIRNL.A	1386.74	2	2.38
		L.PQFEEIRNL.A	1145.60	1	1.52
PI428255-U9_6	Gamma gliadin	F.VQPQPQQL.G	937.51	1	1.69
		L.PQQQLPQQH.L	1103.56	1	1.59
		Y.PQQSLPQQQLPQQH.L	1656.85	2	3.57
		L.GQGM*PM*QPQHQLGQGL.S	1738.81	2	2.21
		F.YQYQQPL.T	939.46	1	1.59
		L.FQQQQPYPQQSLPQQQLPQQH.L	2576.26	2	2.65
PI428255-U9_7	Alpha gliadin	F.RPEQPYPQPQPQ.Y	1464.72	2	2.10
		F.RPEQPYPQPQPQY.S	1627.79	2	2.97
PI428255-U9_8	Alpha gliadin	A.VRVPVPQ.L	794.49	1	1.65
		F.RPQQPYPQPQPQ.Y	1463.74	2	2.89
		F.RPQQPYPQPQPQY.S	1626.80	2	2.01
		F.RPQQPYPQPQPQY.S	1626.80	2	2.45
		H.QQQKQQQQPSSQVSF.Q	1775.87	2	2.16

		L.HQQQKQQQQPSSQVSF.Q	1912.93	2	3.51
		L.HQQQKQQQQPSSQVSF.Q	1912.93	2	3.68
		L.PQFEEIRNL.A	1145.60	1	1.66
		L.QQQLIPCMDVVLQQH.N	1836.94	2	2.21
		Q.TLPAMCN.V	806.38	1	1.52
PI428255-U9_9	Alpha gliadin	A.VRVPVPQ.L	794.49	1	1.61
		A.VRVPVPQLQPQNPSQQEPQEQVPL.V	2735.45	3	3.49
		F.RPQQPYPQPQPQ.Y	1463.74	2	2.11
		F.RPQQPYPQPQPQY.S	1626.80	2	2.70
		I.PCMDVVLQQH.N	1226.60	1	1.81
		L.ALQTLPAMCNVY.I	1380.70	1	1.73
		L.QQQLIPCMDVVL.Q	1443.76	1	1.66
		W.QIPEQSQCQAIHNVVH.A	1887.94	2	2.30
PI428255-U9_10	Alpha gliadin	A.VRVPVPQ.L	794.49	1	1.92
		A.VRVPVPQLQPQNPSQQEPQ.E	2169.14	2	2.07
		F.RPQQPYPQPQPQ.Y	1463.74	2	2.54
		H.NVVHAIIL.H	878.55	1	1.50
		L.ALQTLPAMCNVY.I	1380.70	1	1.69
		L.HQQQKQQQQPSSQVSF.Q	1912.93	2	3.64
		L.QQQLIPCMDVVLQQH.N	1836.94	2	2.46
PI428255-U9_12	Alpha gliadin	L.QQQLIPCMDVVLQQH.N	1836.94	2	2.49
		Y.LQLQPFPKPQQPY.S	1583.86	2	2.83
		Y.LQLQPFPKPQQPY.S	1583.86	2	3.01
		Y.QLLQGL.C	671.41	1	1.72
PI428255-U9_13	Gamma gliadin	F.ASIVAGISGQ.-	902.49	1	1.59
		F.QLVQGQGIIQPQQPAQY.E	1896.00	2	2.62
		H.SIIMQQEQQQGIQ.I	1530.76	2	2.03
		H.SIIMQQEQQQGIQIL.R	1756.93	2	2.42
		L.RTLPNMCNVY.V	1267.62	1	1.80
		L.VLRTLPNM*CNVY.V	1495.78	2	2.93
		L.VLRTLPNMCNVY.V	1479.78	2	2.41
		L.VQGQGIIQPQQPAQY.E	1654.86	2	2.22
		Q.SQQPQQPFPQPQQPQ.Q	1762.85	2	2.89
		Y.EVIRSLVL.R	928.58	1	1.75
		Y.VRPDCSTINAPF.A	1376.69	2	2.71
PI428255-U9_14	Globulin	L.TGRAGEGAVGVPL.F	1183.64	1	2.30
		R.QILEQQL.T	871.49	1	1.67
PI428255-U9_15	Gamma gliadin	F.ASIVAGISGQ.-	902.49	1	1.59
		F.ASIVAGISGQ.-	902.49	1	1.58
		H.SIIMQQEQ.Q	976.48	1	1.65
		L.RTLPNMCNVY.V	1267.62	1	1.80
		Y.VRPDCSTINAPF.A	1376.69	1	2.07
PI428255-U9_16	Avenin-like protein	Q.AQLLEQM*.Q	848.42	1	2.02

Table S5. MOLDI-TOF/TOF identification of the protein spots on 2-DE gels of the glutenin fraction from *T. urartu* accessions.

Spot	Protein	Observed	Mass spectrum
PI428270-U10_4	Alpha/beta-gliadin	903.34	CCQHLW
		1153.54	NPQAQGSVQPQ
		1386.71	QLPQFEEIRNL
		1463.74	RPQQPYPQPQPQ
		1626.80	RPQQPYPQPQPQY
PI428270-U10_5	Alpha/beta-gliadin	1463.65	RPQQPYPQPQPQ
		1626.70	RPQQPYPQPQPQY
		1641.69	QQQPFRPQQPYPQ
PI428270-U10_6	Alpha/beta-gliadin	1463.64	RPQQPYPQPQPQ
		1626.70	RPQQPYPQPQPQY
PI428270-U10_7	Avenin	1035.44	RQQCCRQ
		1118.49	VQERPQQSF
		1231.57	VQERPQQSFL
		1370.58	QQQQQHVDRGF
		1586.71	LRPKTSQQNNCQL
		1626.68	QQQQQQQHVDRGF
		1648.75	HVDRGFVQPQPQQL
		1925.82	AHISEPSRCPAIHNTVH
PI428270-U10_8	Alpha/beta-gliadin	1463.71	RPQQPYPQPQPQ
		1626.77	RPQQPYPQPQPQY
PI428270-U10_9	Alpha/beta-gliadin	903.31	CCQHLW
		1463.66	RPQQPYPQPQPQ
		1523.73	IPEQSQCQAIHNV
		1626.71	RPQQPYPQPQPQY
		1912.82	HQQKKQQQQPSSQVSF
PI428270-U10_10	Alpha/beta-gliadin	1386.67	QLPQFEEIRNL
		1463.67	RPQQPYPQPQPQ
		1626.73	RPQQPYPQPQPQY
		1912.84	HQQKKQQQQPSSQVSF
PI428270-U10_11	Avenin	1098.48	QQRPQQQW
		1438.68	AIRQQRPQQQW
		1499.67	QAQQPAQLESIRM
		1537.71	RCQAIHNVAEAIR
		1851.84	NVAEAIRQQRPQQQW
		2884.11	TVPFPQTPVDQPTSCQNVQHQCCR
PI428270-U10_12	Alpha/beta-gliadin	1386.67	QLPQFEEIRNL
		1463.67	RPQQPYPQPQPQ
		1626.72	RPQQPYPQPQPQY
		1641.72	QQQPFRPQQPYPQ
		1749.78	PSRQNPQAQGSVQPQQ

PI428270-U10_13	Gamma-hordein	825.49	AQIPRQL
		1218.59	ILPRSDCQVM
		1267.58	RTLPNMCNVY
		1376.65	VRPDCSTINAPF
		1423.64	LQQQMNPCKNY
		1479.73	VLRTLPNMCNVY
PI428270-U10_14	Alpha/beta-gliadin	1386.66	QLPQFEEIRNL
		1463.66	RPQQPYPQPQPQ
		1626.71	RPQQPYPQPQPQY
PI428270-U10_15	Avenin	970.47	VQIPEQTR
		1130.48	VQIPEQTRC
		1426.46	SCQNVQSQCCR
		1553.69	RCQAIHNVAESIR
		1776.70	GQCQHHQQSGQQQLL
		2074.88	RCQAIHNVAESIRQQQH
PI428270-U10_16	Avenin	970.47	VQIPEQTR
		1110.48	VQHQCSPVR
		1130.49	VQIPEQTRC
		1211.55	QLVQIPEQTR
		1371.62	QLVQIPEQTRC
		1426.48	SCQNVQSQCCR
		1553.70	RCQAIHNVAESIR
		1776.71	GQCQHHQQSGQQQLL
		2074.90	RCQAIHNVAESIRQQQH
		2823.09	VQHQCSPVRTPFPQTQGEQHSSCQ
		3288.31	VQHQCSPVRTPFPQTQGEQHSSCQTVQH
PI428270-U10_17	Gamma-hordein	825.44	AQIPRQL
		1218.52	ILPRSDCQVM
		1267.51	RTLPNMCNVY
		1376.57	VRPDCSTINAPF
		1423.55	LQQQMNPCKNY
		1479.64	VLRTLPNMCNVY
PI428270-U10_22	Avenin	898.37	RCQAIHN
		970.47	VQIPEQTR
		1110.47	VQHQCSPVR
		1130.49	VQIPEQTRC
		1211.59	QLVQIPEQTR
		1258.57	VQIPEQTRCK
		1371.61	QLVQIPEQTRC
		1426.46	SCQNVQSQCCR
		1667.59	SCQNVQSQCCRQL
		1776.70	GQCQHHQQSGQQQLL
PI428202-U2_4	Gamma-gliadin	1120.45	RQQCCQQL

		1423.59	LQQQMNPCKNY
		1820.83	QIFPQPQQTFPHQPQ
		2389.07	SQQPQQIFPQPQQTFPHQPQ
PI428202-U2_5	Alpha/beta-gliadin	1463.66	RPQQPYPQPQPQ
		1626.71	RPQQPYPQPQPQY
		1982.91	GSVQPQQLPQFEEIRNL
PI428202-U2_11	Alpha/beta-gliadin	1386.66	QLPQFEEIRNL
		1463.66	RPQQPYPQPQPQ
		1626.71	RPQQPYPQPQPQY
PI428255-U9_11	Alpha/beta-gliadin	1386.69	QLPQFEEIRNL
		1463.69	RPQQPYPQPQPQ
		1626.75	RPQQPYPQPQPQY
		1982.97	GSVQPQQLPQFEEIRNL
		2040.92	HQQKKQQQQPSSQVSFQ
		2182.07	AQGSVQPQQLPQFEEIRNL

Table S6. Nucleotide sequence identities of LMW-GS genes in *T. urartu* to the previously reported genes/allelic variants.

Gene and variants	*Glu-A3, B3 & D3*		Taxonomy	Loci	Reference
	Accession No.	Gene/Allele			
TuA3-385a	JX878094(99%)	LMW-GS-D3-385 allele	*Triticum aestivum*	Glu-D3	Zhang et al. 2013
(KM085196)	FJ755311(99%)	D3-4	*Triticum aestivum*	Glu-D3	Dong et al. 2010
	EF437430(99%)	GluDt3-64 allele	*Aegilops tauschii*	Glu-D3	Direct Submission by Zhao and others (2007)
TuA3-385b	FJ755318(99%)	D3-4	*Triticum aestivum*	Glu-D3	Dong et al. 2010
(KM085198)	JX878195 (98%)	LMW-GS-D3-385 allele	*Triticum aestivum*	Glu-D3	Zhang et al. 2013
	EF437430(98%)	GluDt3-64 allele	*Aegilops tauschii*	Glu-D3	Direct Submission by Zhao and others (2007)
TuA3-373	JX878234(99%)	LMW-GS-A3-373 allele	*Triticum aestivum*	Glu-A3	Zhang et al. 2013
(KM065456)	AJ293099(92%)	lmw-gs3	*Triticum durum*		Direct Submission by D'Ovidio
	EF190878(97%)	LMW-m1	*Triticum monococcum*	Glu-A3	Direct Submission by Jiang and others (2006)
TuA3-391	JX878196 (99%)	LMW-GS-A3-391 allele	*Triticum aestivum*	Glu-A3	Zhang et al. 2013
(KM085200)	X51759(94%)		*Triticum durum*		Direct Submission by Cassidy and others (1990)
TuA3-392	JX878196 (98%)	LMW-GS-A3-391 allele	*Triticum aestivum*	Glu-A3	Zhang et al. 2013
(KM085206)	X51759(96%)		*Triticum durum*		Direct Submission by Cassidy and others (1990)
TuA3-397a	FJ549946(96%)	GluA3-5	*Triticum aestivum*	Glu-A3	Direct Submission by wang and others (2008)
(KM085220)	JX878242(96%)	LMW-GS-A3-394b	*Triticum aestivum*	Glu-A3	Zhang et al., 2013
	AJ293099(96%)	lmw-gs3	*Triticum durum*		Direct Submission by D'Ovidio
	FJ441117 (94%)		*Triticum monococcum*		Vaccino et al., 2009
TuA3-397b	JX878221(99%)	A3-400	*Triticum aestivum*	Glu-A3	Zhang et al., 2013
(KM085256)	AJ293099(98%)	lmw-gs3	*Triticum durum*		Direct Submission by D'Ovidio
	FJ441117 (98%)		*Triticum monococcum*		Vaccino et al., 2009
TuA3-400	JX878221(99%)	A3-400	*Triticum aestivum*	Glu-A3	Zhang et al., 2013
(KM085257)	AJ293099(98%)	lmw-gs3	*Triticum durum*		Direct Submission by D'Ovidio

	FJ441117 (98%)		*Triticum monococcum*		Vaccino et al., 2009
TuA3-402	FJ549937 (99%)	GluA3-23	*Triticum aestivum*	Glu-A3	Wang et al., 2010
(KM085231)	JX878097 (99%)	LMW-GS-A3-402b	*Triticum aestivum*	Glu-A3	Zhang et al., 2013
TuA3-460	FJ972196 (90%)		*Triticum aestivum*	Glu-B3	Direct Submission by Han and others (2009)
(KM085237)	EU305551 (89%)		*Aegilops longissima*	Glu-S	Direct Submission by Huang and others (2009)
	JX877942(89%)	LMW-GS-B3-570	*Triticum aestivum*	Glu-B3	Zhang et al., 2013
TuA3-463	FJ972196 (90%)		*Triticum aestivum*	Glu-B3	Direct Submission by Han and others (2009)
(KM085241)	EU305551 (89%)		*Aegilops longissima*	Glu-S	Direct Submission by Huang and others (2009)
	JX877942(89%)	LMW-GS-B3-570	*Triticum aestivum*	Glu-B3	Zhang et al., 2013
TuA3-474	FJ972196 (89%)		*Triticum aestivum*	Glu-B3	Direct Submission by Han and others (2009)
(KM085243)	FJ824796(88%)		*Aegilops longissima*	Glu-S	Direct Submission by Wang and others (2009)
	FJ461690 (88%)		*Triticum dicoccoides*		Direct Submission by Wu and others (2009)
	JX878202 (87%)	LMW-GS-B3-510	*Triticum aestivum*	Glu-B3	Zhang et al., 2013
	FJ824794 (88%)	GluB3-410 allele	*Aegilops speltoides*	Glu-B3	Direct Submission by Wang and others (2009)
TuA3-495	JX877857 (95%)	LMW-GS-A3-502b	*Triticum aestivum*	Glu-A3	Zhang et al., 2013
(KM085244)	AJ293098(94%)	lmw-gs2	*Triticum durum*		Direct Submission by D'Ovidio (2000)
	DQ234068 (91%)		*Triticum monococcum*		Direct Submission by Ma and others (2005)
TuA3-498	JX878133（93%）	LMW-GS-A3-502f	*Triticum aestivum*	Glu-A3	Zhang et al., 2013
(KM085258)	AJ293098(93%)	lmw-gs2	*Triticum durum*		Direct Submission by D'Ovidio (2000)
	DQ234068 (90%)		*Triticum monococcum*		Direct Submission by Ma and others (2005)
TuA3-502a	JX877857 (97%)	LMW-GS-A3-502b	*Triticum aestivum*	Glu-A3	Zhang et al., 2013
(KM085261)	AJ293098(97%)	lmw-gs2	*Triticum durum*		Direct Submission by D'Ovidio (2000)
TuA3-502b	JX877857 (97%)	LMW-GS-A3-502b	*Triticum aestivum*	Glu-A3	Zhang et al., 2013
(KM085246)	AJ293098(97%)	lmw-gs2	*Triticum durum*		Direct Submission by D'Ovidio (2000)
TuA3-502c	JX877857 (98%)	LMW-GS-A3-502b	*Triticum aestivum*	Glu-A3	Zhang et al., 2013

(KM085271)	AJ293098(98%)	lmw-gs2	*Triticum durum*		Direct Submission by D'Ovidio (2000)
	AY146588 (89%)		*Triticum monococcum*		Wicker et al., 2003
TuA3-502d	JX877857 (98%)	LMW-GS-A3-502b	*Triticum aestivum*	Glu-A3	Zhang et al., 2013
(KM085247)	AJ293098(97%)	lmw-gs2	*Triticum durum*		Direct Submission by D'Ovidio (2000)
TuA3-520	JX877857 (95%)	LMW-GS-A3-502b	*Triticum aestivum*	Glu-A3	Zhang et al., 2013
(KM085275)	AJ293098(94%)	lmw-gs2	*Triticum durum*		Direct Submission by D'Ovidio (2000)
	DQ234068 (89%)		*Triticum monococcum*		Direct Submission by Ma and others (2005)
TuA3-590	JX877857 (96%)	LMW-GS-A3-502b	*Triticum aestivum*	Glu-A3	Zhang et al., 2013
(KM085253)	HE647817 (88%)		*Triticum durum*		Direct Submission by Sestili and others (2011)
TuA3-593	JX878099 (94%)	LMW-GS-A3-484 allele	*Triticum aestivum*	Glu-A3	Zhang et al., 2013
(KM085254)	HE647817 (87%)		*Triticum durum*		Direct Submission by Sestili and others (2011)
TuA3-535	JX877797 (94%)	LMW-GS-A3-567-2 allele	*Triticum aestivum*	Glu-A3	Zhang et al., 2013
(KM085278)	FJ441107 (94%)		*Triticum monococcum*		Vaccino et al., 2009
	DQ217661 (93%)	LMW-GS Y5	*Triticum dicoccoides*		Direct Submission by Xiao and others (2005)
TuA3-538a	FJ441107 (95%)		*Triticum monococcum*		Vaccino et al., 2009
(KM085285)	JX877797 (94%)	LMW-GS-A3-567-2 allele	*Triticum aestivum*	Glu-A3	Zhang et al., 2013
	DQ234068 (94%)		*Triticum monococcum*		Direct Submission by Ma and others (2005)
	DQ217661 (94%)	LMW-GS Y5	*Triticum dicoccoides*		Direct Submission by Xiao and others (2005)
TuA3-538b	JX877797 (94%)	LMW-GS-A3-567-2 allele	*Triticum aestivum*	Glu-A3	Zhang et al., 2013
(KM085288)	FJ441107 (95%)		*Triticum monococcum*		Vaccino et al., 2009
	DQ217661 (93%)	LMW-GS Y5	*Triticum dicoccoides*		Direct Submission by Xiao and others (2005)
TuA3-538c	JX877797 (93%)	LMW-GS-A3-567-2 allele	*Triticum aestivum*	Glu-A3	Zhang et al., 2013
(KM085295)	FJ441107 (94%)		*Triticum monococcum*		Vaccino et al., 2009
	DQ217661 (92%)	LMW-GS Y5	*Triticum dicoccoides*		Direct Submission by Xiao and others (2005)
TuA3-538d	FJ441107 (95%)		*Triticum monococcum*		Vaccino et al., 2009

(KM085248)	JX877797 (94%)	LMW-GS-A3-567-2 allele	*Triticum aestivum*	Glu-A3	Zhang et al., 2013
	DQ217661 (93%)	LMW-GS Y5	*Triticum dicoccoides*		Direct Submission by Xiao and others (2005)
TuA3-538e	JX877797 (93%)	LMW-GS-A3-567-2 allele	*Triticum aestivum*	Glu-A3	Zhang et al., 2013
(KM085296)					
TuA3-657	HE647817 (97%)		*Triticum durum*		Direct Submission by Sestili and others (2011)
(KM085321)	JX878192 (96%)	LMW-GS-A3-646b allele	*Triticum aestivum*	Glu-A3	Zhang et al., 2013
TuA3-406	JX877995(90%)	LMW-GS-A3-643 allele	*Triticum aestivum*	Glu-A3	Zhang et al., 2013
(KM085234)	DQ217662 (90%)	LMW-GS Y13	*Triticum dicoccoides*		Direct Submission by Xiao and others (2005)
	DQ234068 (88%)		*Triticum monococcum*		Direct Submission by Ma and others (2005)
TuA3-555	JX878185 (95%)	LMW-GS-A3-573 allele	*Triticum aestivum*	Glu-A3	Zhang et al., 2013
(KM085297)	FJ441107 (94%)		*Triticum monococcum*		Vaccino et al., 2009
	DQ217661 (90%)	LMW-GS Y5	*Triticum dicoccoides*		Direct Submission by Xiao and others (2005)
TuA3-576a	JX878185 (98%)	LMW-GS-A3-573 allele	*Triticum aestivum*	Glu-A3	Zhang et al., 2013
(KM085298)	DQ217661 (93%)	LMW-GS Y5	*Triticum dicoccoides*		Direct Submission by Xiao and others (2005)
	DQ234068 (90%)		*Triticum monococcum*		Direct Submission by Ma and others (2005)
TuA3-576b	JX878185 (97%)	LMW-GS-A3-573 allele	*Triticum aestivum*	Glu-A3	Zhang et al., 2013
(KM085303)	DQ217661 (93%)	LMW-GS Y5	*Triticum dicoccoides*		Direct Submission by Xiao and others (2005)
	DQ234068 (91%)		*Triticum monococcum*		Direct Submission by Ma and others (2005)
TuA3-576c	JX878185 (97%)	LMW-GS-A3-573 allele	*Triticum aestivum*	Glu-A3	Zhang et al., 2013
(KM085304)	DQ217661 (93%)	LMW-GS Y5	*Triticum dicoccoides*		Direct Submission by Xiao and others (2005)
	DQ234068 (90%)		*Triticum monococcum*		Direct Submission by Ma and others (2005)
TuA3-576d	JX878185 (98%)	LMW-GS-A3-573 allele	*Triticum aestivum*	Glu-A3	Zhang et al., 2013
(KM085251)	DQ217661 (93%)	LMW-GS Y5	*Triticum dicoccoides*		Direct Submission by Xiao and others (2005)
	DQ234068 (91%)		*Triticum monococcum*		Direct Submission by Ma and others (2005)
TuA3-576e	JX878185 (98%)	LMW-GS-A3-573 allele	*Triticum aestivum*	Glu-A3	Zhang et al., 2013

(KM085310)	DQ217661 (92%)	LMW-GS Y5	*Triticum dicoccoides*		Direct Submission by Xiao and others (2005)
	DQ234068 (90%)		*Triticum monococcum*		Direct Submission by Ma and others (2005)
TuA3-579a	JX878185 (97%)	LMW-GS-A3-573 allele	*Triticum aestivum*	Glu-A3	Zhang et al., 2013
(KM085312)	DQ217661 (91%)	LMW-GS Y5	*Triticum dicoccoides*		Direct Submission by Xiao and others (2005)
	DQ234068 (89%)		*Triticum monococcum*		Direct Submission by Ma and others (2005)
TuA3-579b	JX878185 (98%)	LMW-GS-A3-573 allele	*Triticum aestivum*	Glu-A3	Zhang et al., 2013
(KM085318)	DQ217661 (92%)	LMW-GS Y5	*Triticum dicoccoides*		Direct Submission by Xiao and others (2005)
	DQ234068 (90%)		*Triticum monococcum*		Direct Submission by Ma and others (2005)
TuA3-597	JX878185 (95%)	LMW-GS-A3-573 allele	*Triticum aestivum*	Glu-A3	Zhang et al., 2013
(KM085320)	DQ217661 (90%)	LMW-GS Y5	*Triticum dicoccoides*		Direct Submission by Xiao and others (2005)
TuA3-669	JX877995 (96%)	LMW-GS-A3-643 allele	*Triticum aestivum*	Glu-A3	Zhang et al., 2013
(KM085322)	HE647817 (95%)		*Triticum durum*		Direct Submission by Sestili and others (2011)
