# Supplementary material for: Composition, variation, expression and evolution of low-molecular-weight glutenin subunit genes in Triticum urartu
Source: BMC Plant Biol. 2015 Feb 28;15:68. doi: 10.1186/s12870-014-0322-3 (PMC4364320; doi:10.1186/s12870-014-0322-3)

ADDITIONAL FILE 1

Composition, variation, expression and evolution of low-molecular-weight glutenin subunit gene family in *Triticum urartu*

Guangbin Luo[1, 2], Xiaofei Zhang[1,*], Yanlin Zhang[3], Wenlong Yang[1], Yiwen Li[1], Jiazhu Sun[1], Kehui Zhan[3], Aimin Zhang[1, 3, 4], Dongcheng Liu[1, 4]

Figure S1. Sequence alignments of the *TuA3-385* gene identified in *T. urartu* and its homologs in common wheat and *Ae. tauschii*.

Figure S2. Sequence alignments of the *TuA3-391* gene identified in *T. urartu*.

Figure S3. Sequence alignments of the *TuA3-397* and *TuA3-400* genes identified in *T. urartu*.

Figure S4. Sequence alignments of the *TuA3-502* gene identified in *T. urartu*.

Figure S5. Sequence alignments of the *TuA3-538* gene identified in *T. urartu*.

Figure S6. Sequence alignments of the *TuA3-576* gene identified in *T. urartu*.

Figure S7. Sequence alignments of the *TuA3-460* gene identified in *T. urartu*.

Figure S8. Sequence alignments of the deduced proteins of all active LMW-GS genes in *T. urartu*.

Figure S9. Protein sequence alignments of variants of *TuA3-460* gene with s- and m-type genes characterized previously at the *Glu-B3* and *Glu-D3* loci in common wheat and m-type genes in *T. urartu.*

Figure S1. Sequence alignments of the *TuA3-385* gene identified in *T. urartu* and its homologs in common wheat and *Ae. tauschii*. *D3-385* (JX878094) from common wheat, and *GluDt3-64* (EF437430) from *Ae. tauschii*.

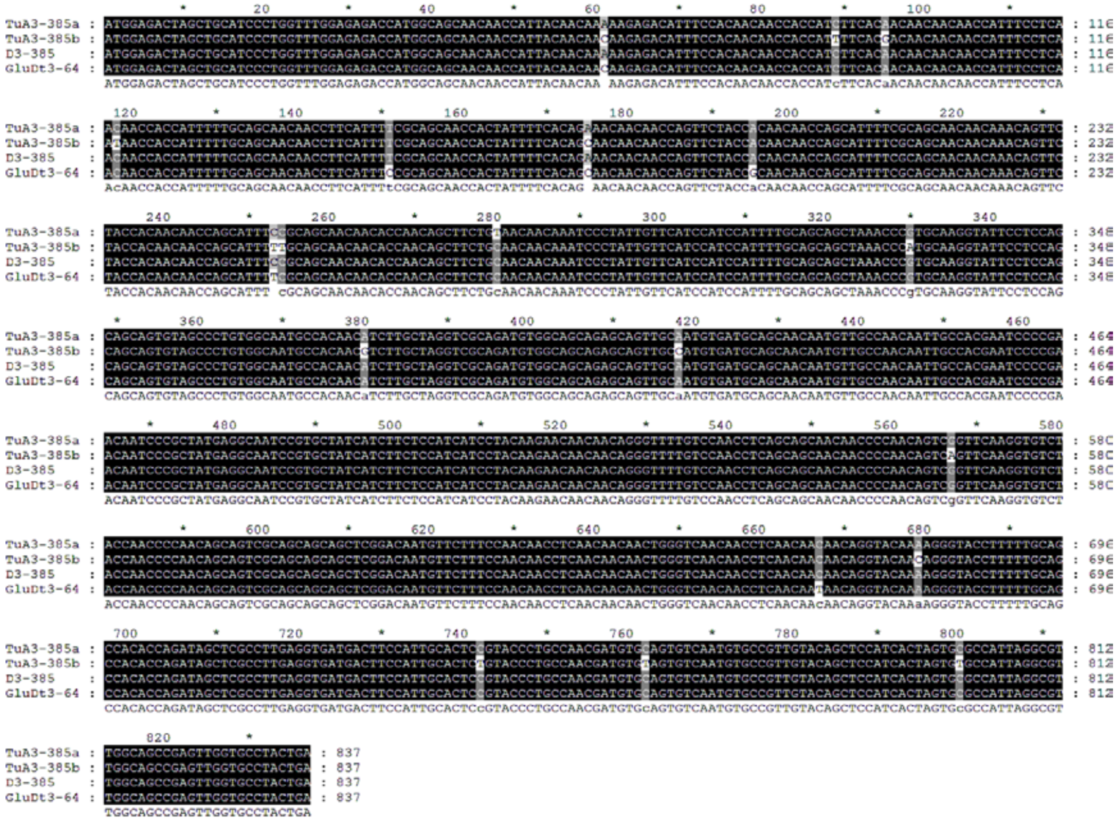

Figure S2. Sequence alignments of the *TuA3-391* gene identified in *T. urartu*.

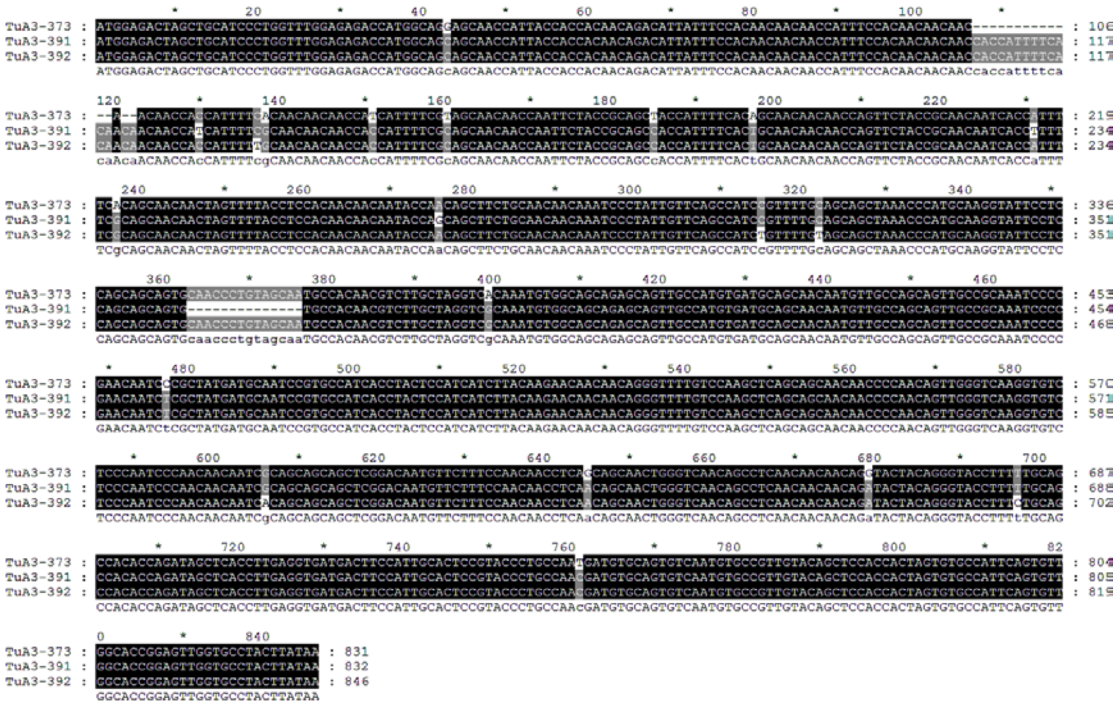

Figure S3. Sequence alignments of the *TuA3-397* and *TuA3-400* genes identified in *T. urartu*.

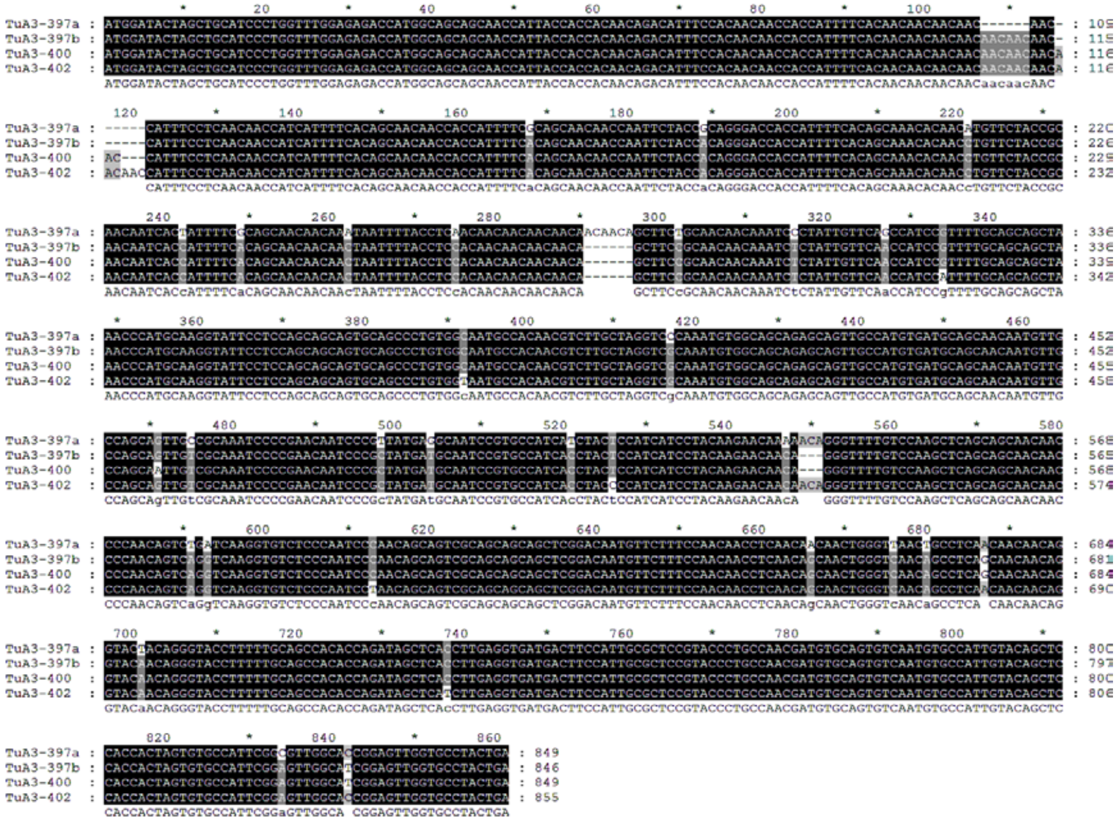

Figure S4. Sequence alignments of the *TuA3-502* gene identified in *T. urartu*.

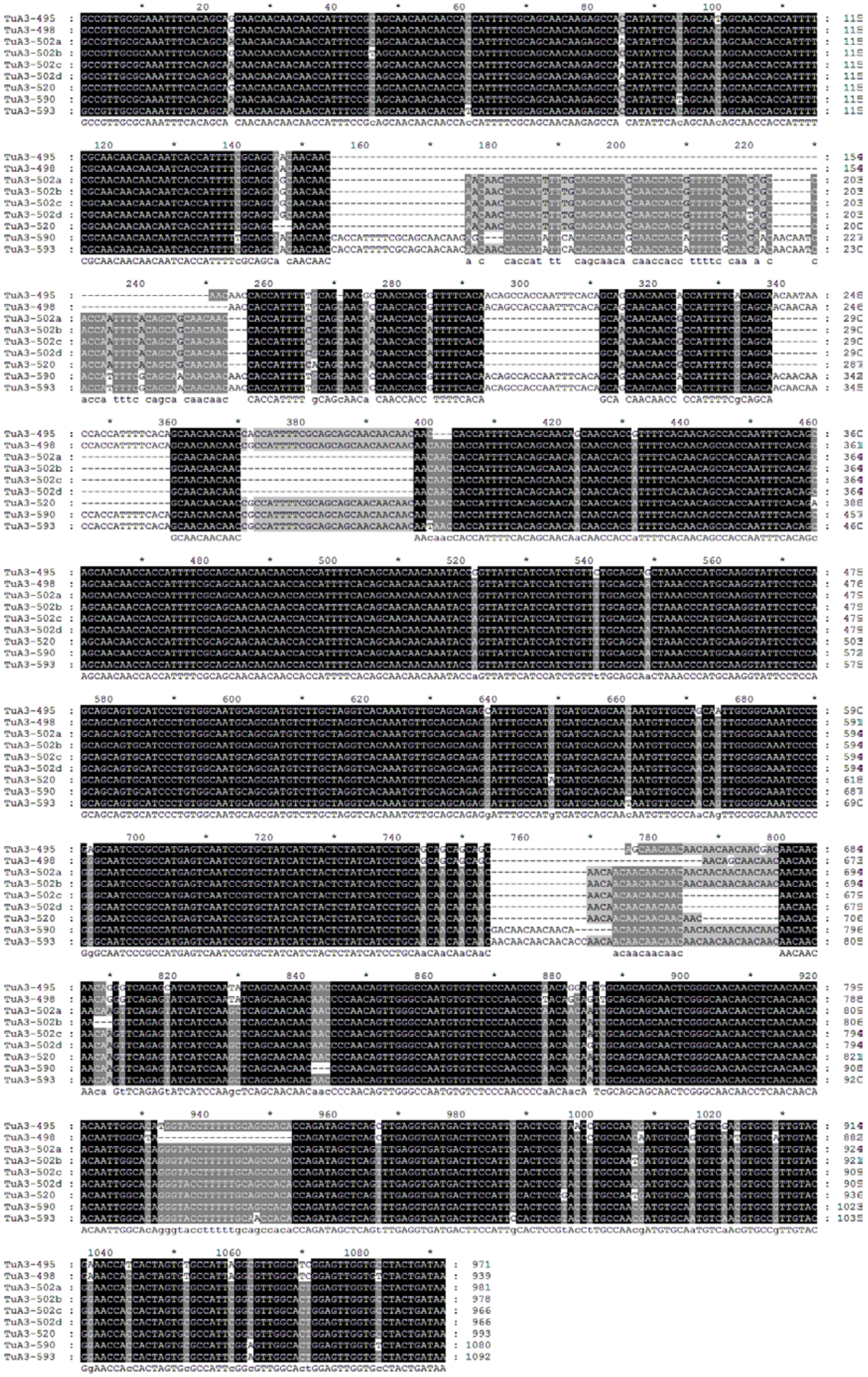

Figure S5. Sequence alignments of the *TuA3-538* gene identified in *T. urartu*.

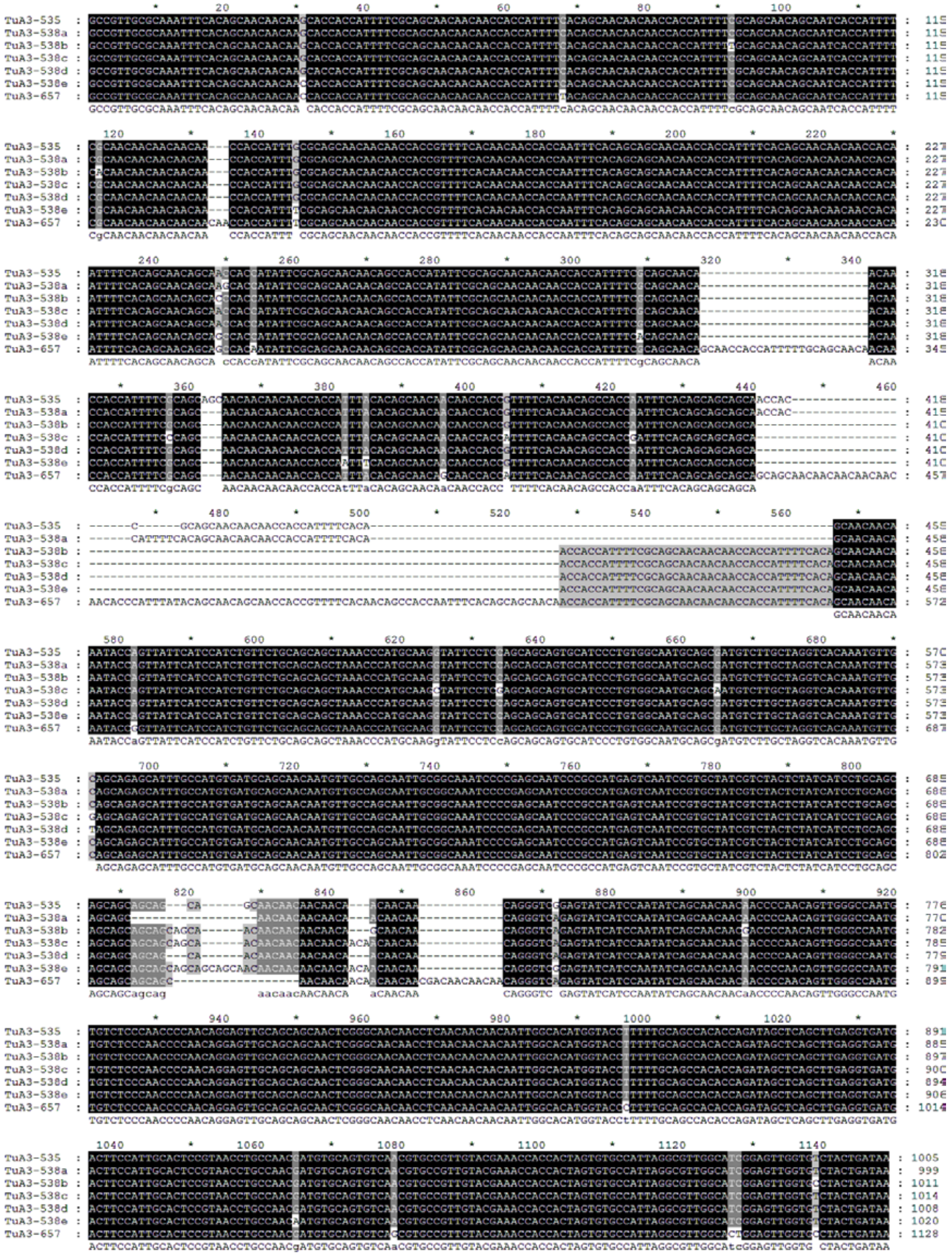

Figure S6. Sequence alignments of the *TuA3-576* gene identified in *T. urartu*.

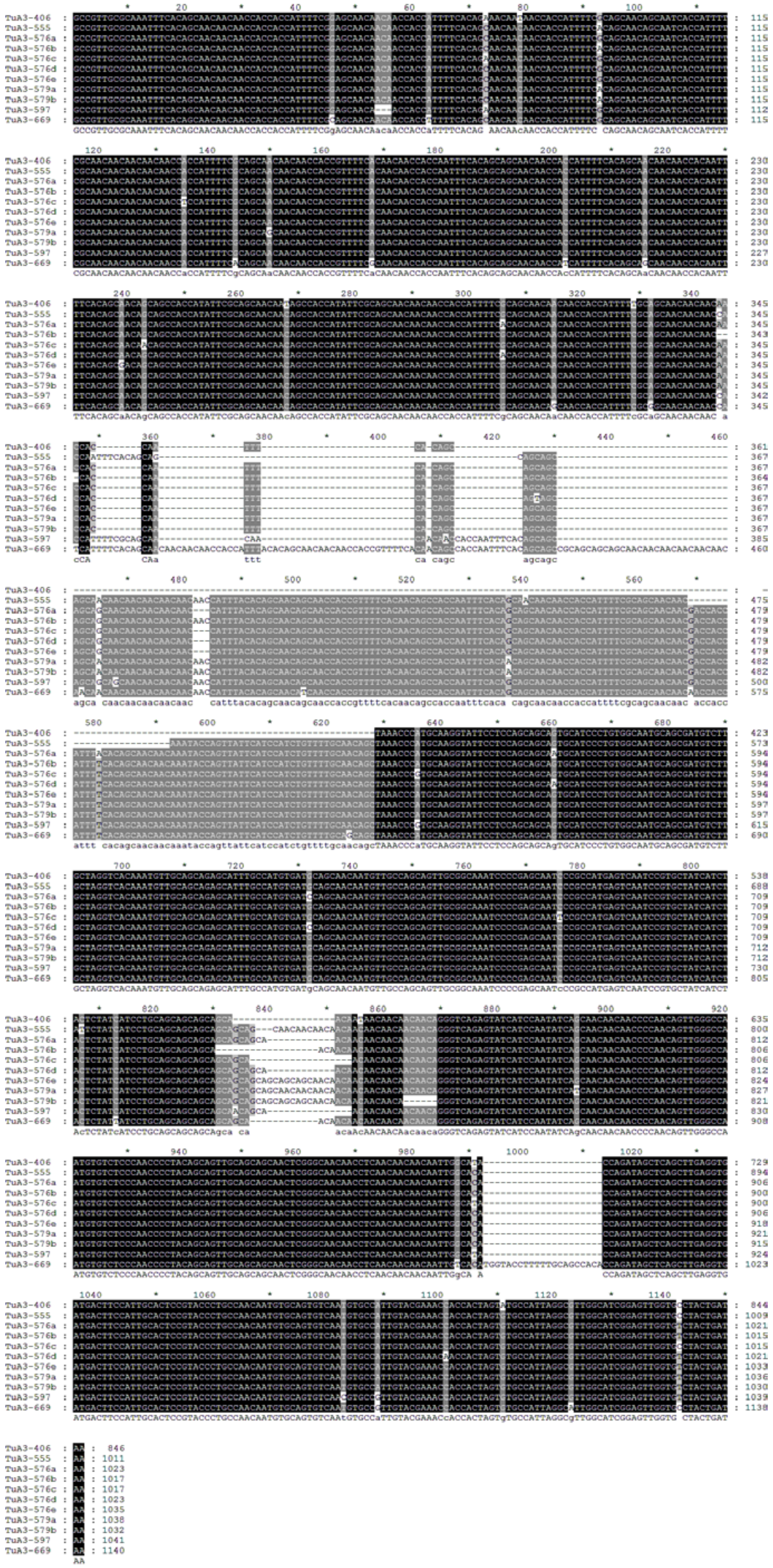

Figure S7. Sequence alignments of the *TuA3-460* gene identified in *T. urartu*.

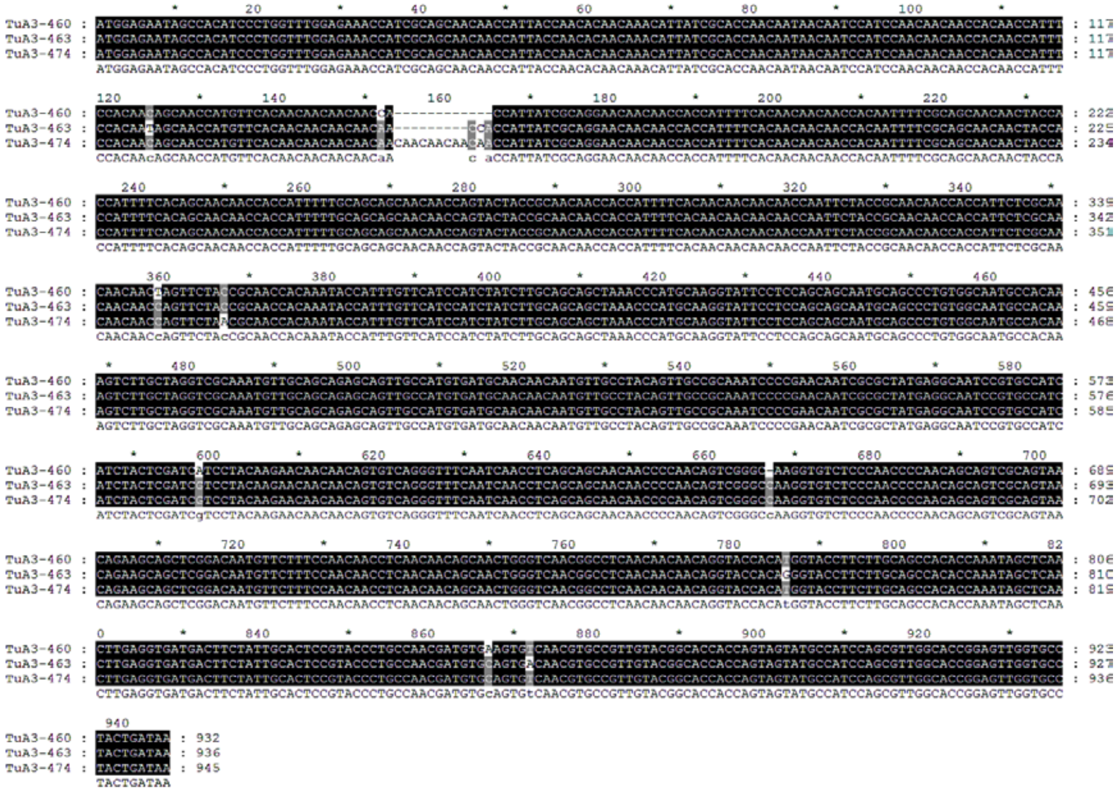

Figure S8. Sequence alignments of the deduced proteins of all active LMW-GS genes in *T. urartu*. Amino acids with green background are cysteine residues.

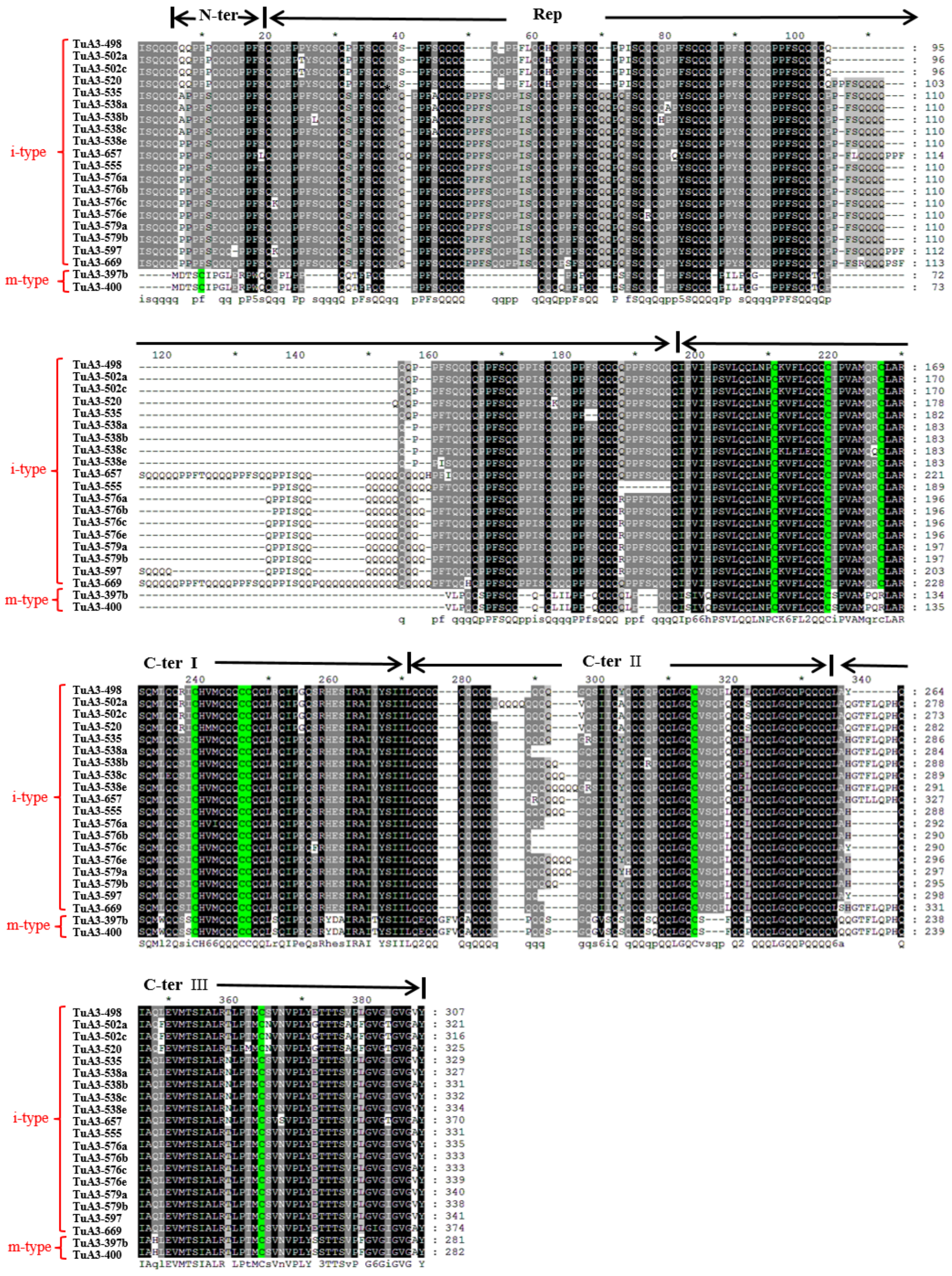

Figure S9. Protein sequence alignments of variants of *TuA3-460* gene with s- and m-type genes characterized previously at the *Glu-B3* and *Glu-D3* loci in common wheat and m-type genes in *T. urartu*. Unique amino acids in TuA3-460 are labeled with red triangles, amino acids shared with s-type LMW-GS genes are labeled with purple triangles, and amino acids shared with m-type LMW-GS genes are labeled with blue triangle. Deletions or insertions are underlined. Cysteine residues are in green background. s-type genes: *B3-544* (JX877787), *B3-578a* (JX878213), *B3-621* (JX878089), *B3-688c* (FJ755309), *B3-813* (JX877882), *D3-578a* (JX877929); m-type genes:, *B3-530a* (JX877786), *B3-548* (JX878004), *B3-570* (JX877942), *D3-385a* (JX878079), *D3-393* (FJ755312), *D3-394* (JF339174), *D3-441* (JF339160), *D3-525* (JF339162), *D3-575* (FJ755310), *D3-586* (JX878008).

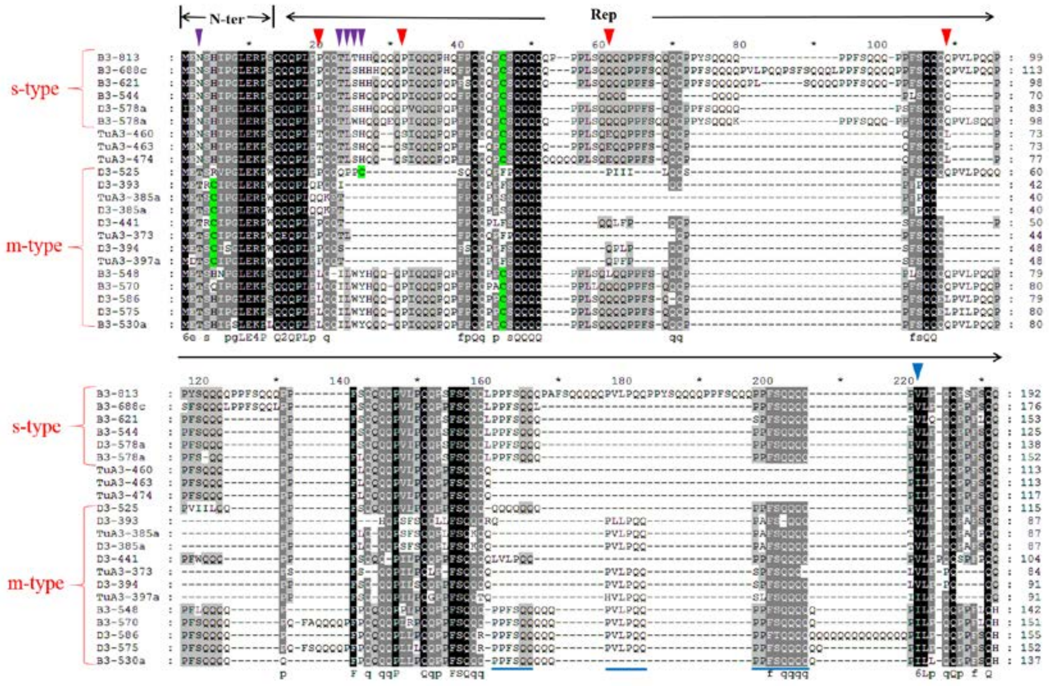

Figure S9 (cont.). Protein sequence alignments of variants of *TuA3-460* gene with s- and m-type genes characterized previously at the *Glu-B3* and *Glu-D3* loci in common wheat and m-type genes in *T. urartu*.

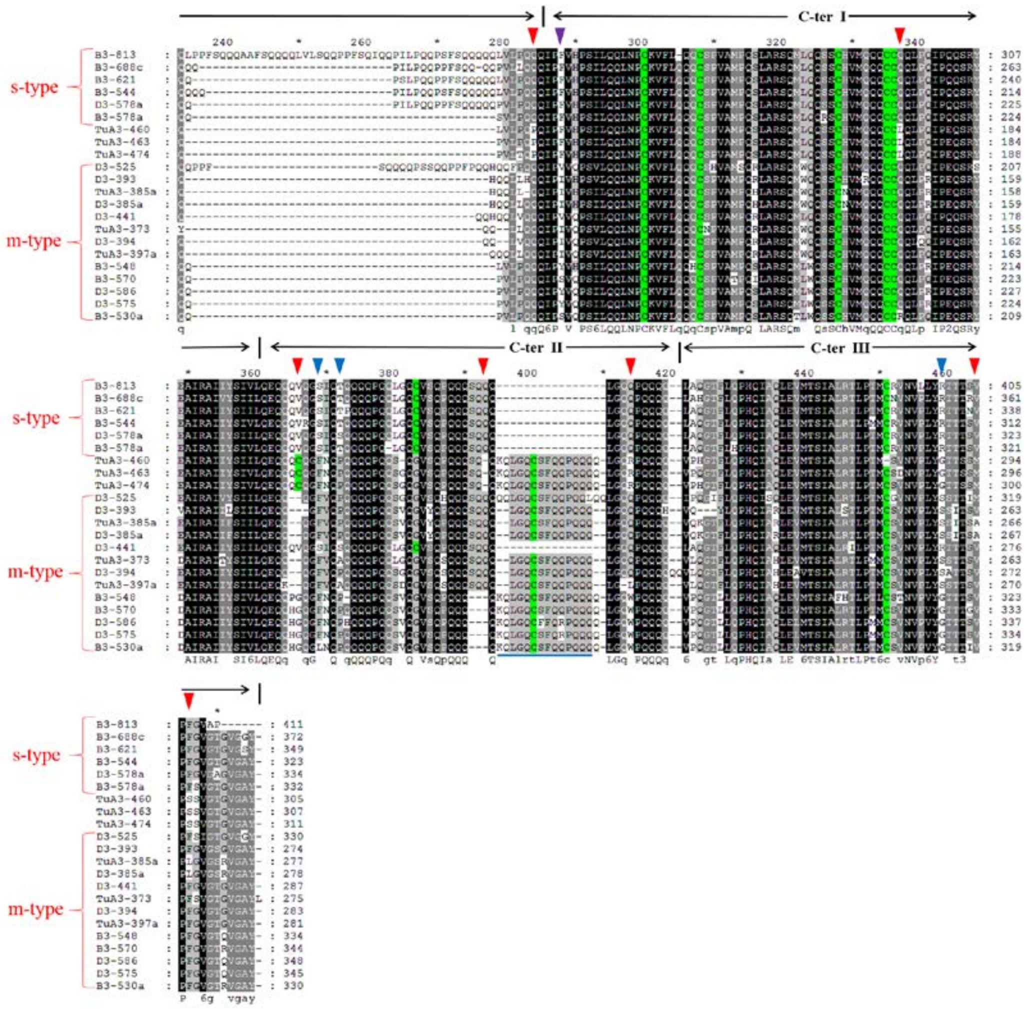

Supplement: Additional file 2: Figure S1. — Sequence alignments of the TuA3-385 gene identified in T. urartu and its homologs in common wheat and Ae. tauschii. Figure S2. Sequence alignments of the TuA3-391 gene identified in T. urartu. Figure S3. Sequence alignments of the TuA3-397 and TuA3-400 genes identified in T. urartu. Figure S4. Sequence alignments of the TuA3-502 gene identified in T. urartu. Figure S5. Sequence alignments of the TuA3-538 gene identified in T. urartu. Figure S6. Sequence alignments of the TuA3-576 gene identified in T. urartu. Figure S7. Protein sequence alignments of variants of TuA3-460 gene with s- and m-type genes characterized previously at the Glu-B3 and Glu-D3 loci in common wheat and m-type genes in T. urartu. Figure S8. Sequence alignments of the TuA3-460 gene identified in T. urartu. Figure S9. Sequence alignments of the deduced proteins of all active LMW-GS genes in T. urartu. [file 12870_2014_322_MOESM2_ESM.pdf]
